# Pak4-mediated crosstalk between necroptotic macrophages and tendon stem/progenitor cells contributes to traumatic heterotopic ossification formation

**DOI:** 10.1038/s41413-025-00463-8

**Published:** 2025-10-20

**Authors:** Ziyang Sun, Hang Liu, Yi Xu, Qian Chen, Gang Luo, Zhengqiang Yuan, Zhenyu Chen, Kuangyu He, Cunyi Fan, Juehong Li, Hongjiang Ruan

**Affiliations:** 1 Department of Orthopedics, Shanghai Sixth People’s Hospital Affiliated to Shanghai Jiao Tong University School of Medicine, Shanghai, PR China; 2 Shanghai Engineering Research Center for Orthopaedic Material Innovation and Tissue Regeneration, Shanghai, PR China

**Keywords:** Homeostasis, Pathogenesis

## Abstract

The formation of traumatic heterotopic ossification (HO) is an abnormal repair process after soft tissue injury. Recent studies establish the involvement of immune cells and cellular metabolism in the tissue healing process; however, their role in HO remains unknown. Here, by using murine burn/tenotomy model in vivo and tendon stem/progenitor cells (TSPCs) osteogenic differentiation model in vitro, together with techniques including transgenic knockout, gene knockdown, transcriptome and proteome sequencings, mass spectrometry, co-immunoprecipitation, seahorse, etc., we reveal a novel p21-activated kinase 4 (Pak4) mediated crosstalk where the necroptotic macrophages arouse TSPCs with reduced fatty acid β-oxidation (FAO), to promote aberrant osteogenic differentiation during HO formation. Necroptosis blockade with *Mlkl* knockout (*C57BL/6JGpt-Mlkl*^*em1Cd1679*^*/Gpt*) significantly reduces HO than WT mice. Extracellular vesicle (EVs) secreted from necroptotic bone marrow-derived macrophages (BMDMs, NecroMφ-EVs) are determined to motivate FAO reduction in TSPCs and result in higher osteogenic activity. *Pak4* conditional knockout (*C57BL/6JGpt-Pak4*^*em1Cflox*^*/Gpt*) in macrophage significantly increases FAO and reduces HO than Flox mice, as well as local injection of *PAK4*^*−/−*^*-*EVs (NecroMφ-EVs with *Pak4* knockout) than NecroMφ-EVs, and the protective effects are reversed after transfection of *Fabp3*^*S122D*^, a phosphomimetic mutant of S122 on fatty acid binding protein 3 (Fabp3) phosphorylation site. Mechanically, after soft tissue injury, macrophages infiltrate, and necroptosis occurs, accompanied by paracrine EVs-derived Pak4, which binds directly to Fabp3 and phosphorylates it at the S122 site in TSPCs, results in reduced FAO, finally osteogenic behavior, and HO formation. This study adds perceptiveness into abnormal regeneration-based theory for traumatic HO and raises treatment strategy development.

## Introduction

Traumatic heterotopic ossification (HO) is a pathological condition characterized by the formation of mature lamellar bone in soft tissues that are outside the skeletal system.^1^ HO is typically triggered by combat-related injuries, as well as fractures, dislocations, and severe burns. The presence of HO leads to considerable limb dysfunction, which significantly impacts quality of life and imposes substantial health and economic burdens on both individuals and society. Although non-steroidal anti-inflammatory drugs (NSAIDs) are commonly employed for the prevention of HO, the incidence remains alarmingly high following trauma, reaching up to 40%.^2^ Therefore, it is imperative to achieve a comprehensive understanding of the complex mechanisms underlying HO formation and to explore effective preventive strategies.

Classically, the process of HO formation comprises three critical components: osteogenic microenvironment, inducible factors, and osteogenic precursor cells.^3^ Immune cells play significant roles in shaping the osteogenic microenvironment and can initiate abnormal healing processes within soft tissues, which have become emerging targets in recent HO research.^4^ Among these immune cells, macrophages, serving as sentinel orchestrators of immune activity and homeostasis, exhibit a close relationship with HO formation.^5^ In murine models of traumatic HO (burn/tenotomy), macrophages are observed early at day 3, peaking at day 7, and persisting for up to 2 weeks; notably, depletion of macrophages results in a more than 50% reduction in HO volume.^6^

Necroptosis, a recently identified cell death pathway that regulates homeostasis and inflammation, is characterized as a type of necrosis mediated by death receptors such as tumor necrosis factor (TNF)-α receptor 1, receptor-interacting protein kinase (RIPK)1, and RIPK3. These receptors initiate the phosphorylation of mixed lineage kinase domain-like (MLKL) proteins, thereby disrupting cellular integrity.^7^ Necroptosis has significant proinflammatory effects, creating a self-perpetuating cycle between regulated cell death (RCD)-driven inflammation and inflammation-induced RCD.^8^ This interaction contributes to acute inflammatory injuries and their tissue repair processes, including acute kidney injury,^9^ myocardial infarction,^10^ liver failure,^11^ and pancreatitis.^12^ For instance, in acute lung injury (ALI), necroptosis activates the RIPK1-RIPK3-MLKL axis to trigger NF-κB/p38 signaling, amplifying proinflammatory cytokine production. This reciprocal enhancement between necroptosis and inflammation accelerates ALI progression.^13^ Indeed, trauma-induced HO exhibits similar pathological features with four histological phases: inflammation, chondrogenesis, osteogenesis, and maturation.^14^ Tnf-α levels are also elevated both in serum plasma and local tissues of burn/tenotomy mice, the initiating factor for necroptosis.^15^ Given the centrality of inflammatory priming in both necroptosis-related injuries and HO initiation, we hypothesize that necroptosis may mechanistically contribute to HO pathogenesis. Moreover, extracellular vesicle (EVs) are lipid bilayer particles secreted by cells that encapsulate RNAs, proteins, lipids, carbohydrates, and metabolites.^16^ Numerous studies indicate that EVs derived from necroptotic cells play pivotal roles in intercellular signaling as well as the exchange and transport of cellular components between cells.^17^

Accumulating evidence indicates that stem cells possess the capacity for self-renewal and multidirectional differentiation in the context of tissue repair.^18^ In cases of tendon injury, tendon stem/progenitor cells (TSPCs) congregate within damaged tissues while simultaneously undergoing normal differentiation to facilitate healing. However, under local inflammatory and osteogenic microenvironments, TSPCs, typically characterized by the expression of platelet-derived growth factor receptor alpha (PDGFRα), transition to exhibit osteogenic behavior, functioning as osteogenic precursor cells that contribute to the pathological basis for HO formation.^19,20^ Nevertheless, the mechanisms underlying the osteogenic differentiation of TSPCs during HO formation remain inadequately understood.

In this study, we aimed to assess the effects of macrophage necroptosis on the aberrant osteogenic healing of TSPCs during HO formation following soft tissue injury. Furthermore, we elucidated the underlying mechanisms involved in the interaction between necroptotic macrophages and affected TSPCs, as well as the intrinsic pathways associated with this process.

## Results

### Macrophage necroptosis was found in tendon lesions in burn/tenotomy mice

**Fig. 1:**
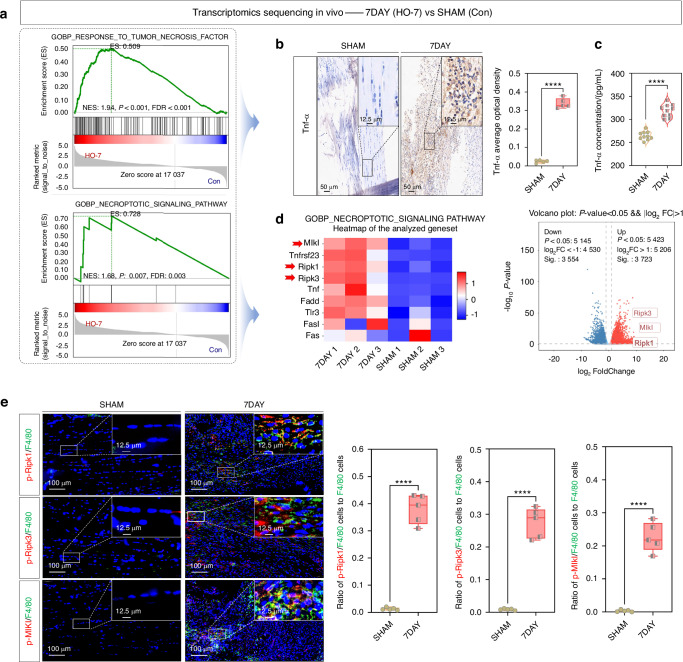
Macrophage necroptosis was found in tendon lesions in burn/tenotomy mice at 7 days post-injury. **a** High-throughput whole-transcriptome sequencing was performed and showed by GSEA for “response to tumor necrosis factor” and “necroptotic signaling pathway” between sham group and the tendon lesions at 7 days; *n* = 3. **b** IHC staining was used to detect the expression of Tnf-α in the sham group and tendon lesions at 7 days; *n* = 5, ****P < 0.000 1, scale bar = 50 μm (original magnification) and 12.5 μm (insert magnification of the boxed area, 4.0x). **c** ELISA was used to detect the concentration of Tnf-α in the sham group and tendon lesions at 7 days; *n* = 10, ****P < 0.000 1. **d** High-throughput whole-transcriptome sequencing was performed and showed by heatmap and volcano plot for necroptotic indicators between sham group and the tendon lesions at 7 days; *n* = 3. **e** IF staining was used to detect the positive cells of p-Ripk1, p-Ripk3 and p-Mlkl (red), co-localized with F4/80 (green), between the sham group and tendon lesions at 7 days; *n* = 5, ****P < 0.000 1, scale bar = 100 μm (original magnification) and 12.5 μm (insert magnification of the boxed area, 4.0x)

A mouse burn/tenotomy model was established in accordance with the relevant literatures (Fig. S1A),^21^ and sham surgery was served as the control group. To investigate the underlying mechanisms of HO, high-throughput whole transcriptome sequencing was conducted to compare the control group with tendon lesions at 7 days post-injury. Gene set enrichment analysis (GSEA) revealed that the “response to tumor necrosis factor” and the “necroptotic signaling pathway” were significantly enriched in burn/tenotomy mice, compared to the control group (Fig. 1a). This finding was further supported by an elevated expression and concentration of Tnf-α, as confirmed by IHC staining (Fig. 1b) and ELISA (Fig. 1c). Both the heatmap and volcano plot (Fig. 1d), along with WB analysis (Fig. S1B), further demonstrated elevated levels of p-Ripk1, p-Ripk3, and p-Mlkl at 7 days post-injury. Moreover, as one of the most crucial immune cells involved in HO formation, macrophages were observed to undergo necroptosis during the early stages of inflammation. This was confirmed through IF staining for p-Ripk1, p-Ripk3, and p-Mlkl, which co-localized with F4/80, a specific marker used for detecting macrophages (Fig. 1e). Similarly, necroptotic macrophages were also detected and confirmed at 3 weeks post-surgery (Fig. S1C-G). In light of the higher level of necroptosis observed in macrophages at 7 days compared to 3 weeks post tendon injury, we selected 7 days as the time point for subsequent analysis of necroptotic macrophages in the HO model.

### Macrophage necroptosis contributed to aberrant osteogenic induction of TSPCs in vitro and traumatic HO formation in vivo

**Fig. 2:**
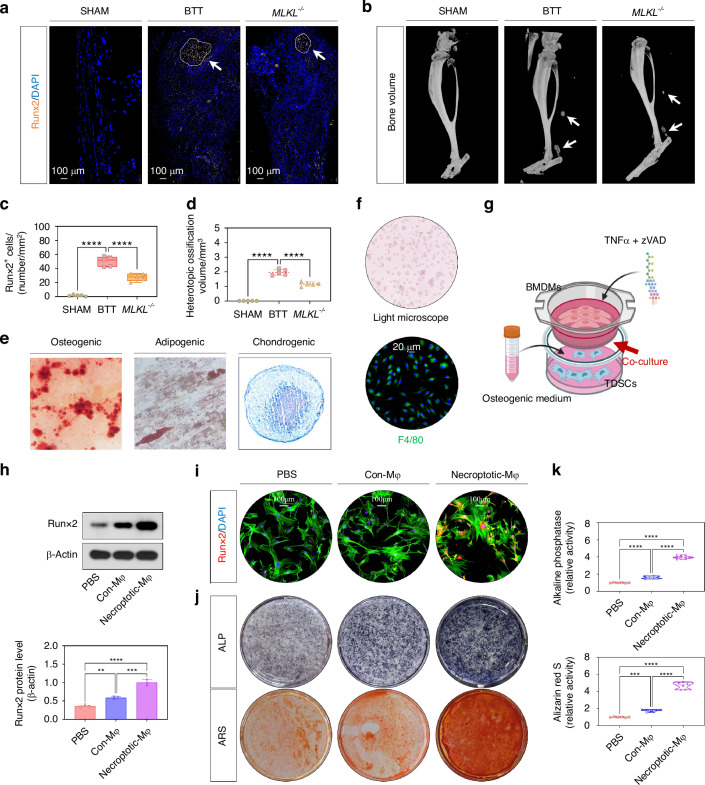
Macrophage necroptosis contributed to aberrant osteogenic induction of TSPCs in vitro and traumatic HO formation in vivo. **a**, **c** IF staining was used to detect osteogenesis by the positive cells of Runx2 (orange) among the sham group and tendon lesions at 3 weeks of WT mice and *Mlkl* KO mice; *n* = 5, scale bar = 100 μm (original magnification), ****P < 0.000 1. **b**, **d** Micro-CT was used to detect HO formation among the sham group and tendon lesions at 10 weeks of WT mice and *Mlkl* KO mice; the volume of HO was quantified from each group; *n* = 5, ****P < 0.000 1. **e** ARS, Oil Red O, and Alcian blue staining were used to detect TSPCs osteogenic, adipogenic, and chondrogenic differentiation, respectively. **f** Light microscope and IF staining for F4/80 were used to identify BMDMs. **g** Schematic depiction of the co-culture model with necroptosis-induced BMDMs and TSPCs with osteogenic induction. **h** WB analysis was used to detect the expression of Runx2 in the osteogenic induced TSPCs, in addition of PBS, Con-Mφ, or Necroptotic-Mφ; *n* = 3, **P < 0.01, ***P < 0.001, ****P < 0.000 1. **i** IF staining was used to detect the expression of Runx2 (red), co-stained with phalloidin (green) and DAPI (blue), in the osteogenic induced TSPCs in addition of PBS, Con-Mφ, or Necroptotic-Mφ; *n* = 6, scale bar = 100 μm. **j**, **k** ALP and ARS staining were used to detect the osteogenesis of TSPCs in addition of PBS, Con-Mφ, or Necroptotic-Mφ; *n* = 6, ***P < 0.001, ****P < 0.000 1

Now that we have confirmed the presence of necroptotic macrophages in the early osteogenic microenvironment, we aimed to determine the effects of cellular necroptosis on trauma-induced HO formation in vivo. To accomplish this, we evaluated mature HO and osteogenic indicators between wild-type (WT) mice and *Mlkl*^−/−^ mice (general knockout models deficient in the key necroptosis mediator protein) using a consistent burn/tenotomy model. IF staining for Runx2 (Fig. 2a, c), SOFG staining (Fig. S2A), micro-CT (Fig. 2b, d), and H&E staining (Fig. S2B) all demonstrated that depletion of *Mlkl* resulted in a significant reduction of osteogenic behavior at 3 weeks post-injury, as well as diminished maturation of HO at 10 weeks following tendon injury.

In light of the fact that the effects of macrophages are primarily mediated through their paracrine functions, we investigated whether the secretome from necroptotic macrophages contributed to the osteogenic potential of TSPCs. TSPCs (Fig. 2e and Fig. S2C, E) and BMDMs (Fig. 2f and Fig. S2D) were isolated and characterized. The coculture of BMDMs and TSPCs was established utilizing the Transwell system (Fig. 2g). The induction of macrophage necroptosis was achieved through stimulation with TNFα in conjunction with zVAD-fmk, which was confirmed by IF staining and WB for p-Mlkl (Fig. S2F, G). TSPCs were also cultured in an osteo-inductive medium. The results demonstrated that following the induction of necroptosis in BMDMs, the osteogenic activity of TSPCs co-cultured with necroptotic BMDMs was significantly greater than that co-cultured with phosphate-buffered saline (PBS)&dimethyl sulfoxide (DMSO)-stimulated BMDMs. This finding was substantiated by a marked increase in WB analysis (Runx2, Opn, and Ocn; Fig. 2h and Fig. S2H), IF staining (Runx2, Opn, and Ocn; Fig. 2i and Fig. S2I), as well as ALP and ARS staining (Fig. 2j, k). The aforementioned results suggested that macrophage necroptosis enhanced trauma-induced HO by augmenting the osteogenic potential of TSPCs. Given that the secretome of macrophages could be primarily categorized into soluble fractions (SFs, conditioned medium devoid of EVs) and EVs, subsequent experiments aimed to investigate their respective roles in the osteogenic behavior of TSPCs and to determine which component exerted a more significant influence.

### Necroptotic macrophages derived EVs contributed to the osteogenic behavior of TSPCs in vitro and traumatic HO formation in vivo

**Fig. 3:**
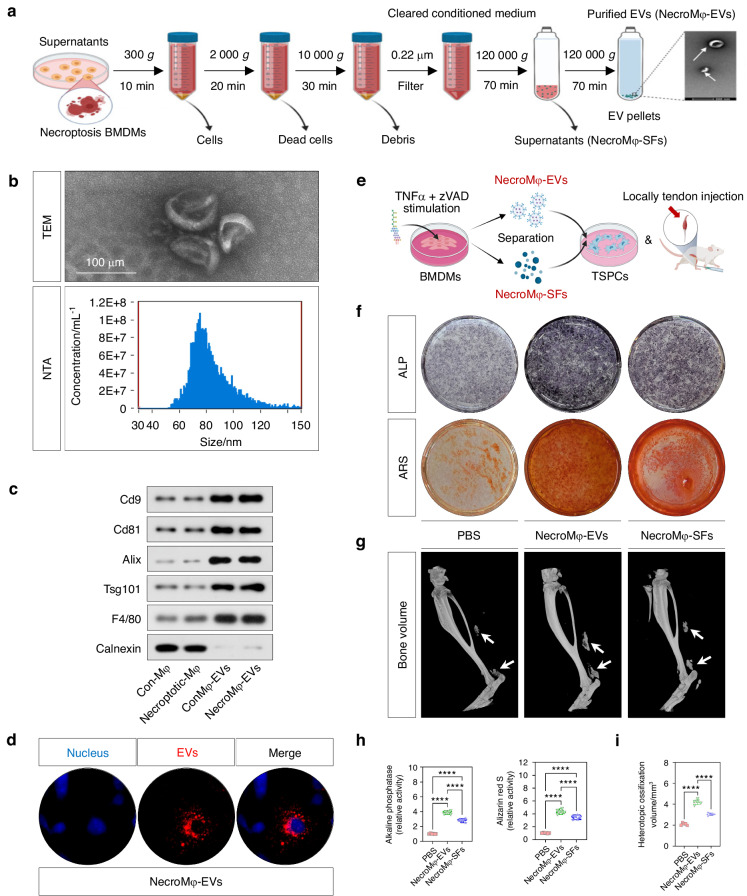
Necroptotic macrophages derived EVs contributed to the osteogenic behavior of TSPCs in vitro and traumatic HO formation in vivo. **a** Schematic depiction of the isolation of necroptotic macrophages derived EVs and SFs (NecroMφ-EVs, NecroMφ-SFs). **b** Transmission electron microscopic (TEM) was used for EVs observation. Nanoparticle tracking analysis (NTA) was used for particle size determination of EVs. **c** WB analysis was used for identification of the EVs positive markers including Cd9, Cd81, Alix, Tsg101, negative marker Calnexin, and parental cell marker F4/80. **d** IF staining was used for checking of EVs internalization in TSPCs. Red indicated Dil-labeled EVs, blue indicated DAPI. **e** Schematic depiction of adding NecroMφ-EVs or NecroMφ-SFs into TSPCs osteogenic medium. **f**, **h** ALP and ARS staining were used to detect the osteogenesis of TSPCs in addition of PBS, NecroMφ-EVs, or NecroMφ-SFs; *n* = 6, ****P < 0.000 1. **g**, **i** Micro-CT was used to detect HO formation in the tendon lesions at 10 weeks in addition of PBS, NecroMφ-EVs, or NecroMφ-SFs; the volume of HO was quantified from each group; *n* = 5, ****P < 0.000 1

After the induction of necroptosis in BMDMs, EVs and SFs derived from necroptotic macrophages (NecroMφ-EVs, NecroMφ-SFs) were isolated (Fig. 3a). Characterization of the isolated EVs was achieved through TEM, NTA, and WB analysis. TEM successfully captured membrane-bound, cup-shaped nanoparticles that conformed to the typical morphology associated with EVs (Fig. 3b). NTA measured the particle size of the isolated EVs, revealing a distribution within the range of 30–150 nm (Fig. 3b). WB analysis confirmed the abundant expression of EV markers, including Cd9, Cd81, Alix, and Tsg101, alongside their parental cell marker F4/80. In contrast, there was bare expression of Calnexin, an endoplasmic reticulum marker (Fig. 3c). This collective data demonstrated the successful isolation of macrophage-derived EVs. Furthermore, we validated the uptake of these EVs by TSPCs. The intracellular space within TSPCs exhibited localization of Dil-labeled EVs, indicating effective internalization regardless of their origin (Fig. 3d).

To investigate how the secretome derived from necroptotic macrophages influenced the osteogenic behavior of TSPCs, we separately incorporated NecroMφ-EVs and NecroMφ-SFs into the osteo-inductive medium in vitro, as well as performed local tendon injections using a burn/tenotomy model in vivo (Fig. 3e). In vitro, the addition of NecroMφ-EVs or NecroMφ-SFs resulted in enhanced osteogenic activity compared to the addition of PBS, as demonstrated by WB analysis (Runx2, Opn, and Ocn; Fig. S3A), IF staining (Runx2, Opn, and Ocn; Fig. S3B), as well as ALP and ARS staining (Fig. 3f, h). In vivo studies revealed that, compared to PBS injection, the levels of mature HO and osteogenic indicators were significantly elevated following the injection of additional NecroMφ-EVs or NecroMφ-SFs. Notably, the enhancement effects were markedly greater with NecroMφ-EVs. This was corroborated by IF staining (Runx2; Fig. S3C), SOFG staining (Fig. S3D), micro-CT (Fig. 3g, i), and H&E staining (Fig. 3). All these findings suggested that NecroMφ-EVs played a significant role in the paracrine effects of macrophage necroptosis.

Moreover, to additionally further explore the route by which NecroMφ-EVs are internalized into TSPCs, it was monitored after treatment with inhibitors. The cellular internalization of EVs primarily occurs through three major endocytic pathways: clathrin-dependent endocytosis, caveolae-dependent endocytosis, and macropinocytosis.^22^ After incorporating NecroMφ-EVs into the osteo-inductive medium of TSPCs in vitro, we employed pharmacological inhibitors targeting each pathway: pitstop 2 (clathrin inhibitor), nystatin (caveolae inhibitor), and EIPA (macropinocytosis inhibitor). Results showed that both pitstop 2 and EIPA significantly reduced the osteogenic activity compared to the addition of DMSO, while nystatin treatment showed only marginal inhibition without statistical significance, as demonstrated by ALP and ARS staining (Fig. S3F). Similar trends for mature HO were also found after incorporating NecroMφ-EVs and each pharmacological inhibitor in vivo (DMSO as control, pitstop 2, nystatin, and EIPA), as demonstrated by micro-CT (Fig. S3G). These findings revealed that the cellular internalization of NecroMφ-EVs in TSPCs predominantly occurred through clathrin-dependent endocytosis and macropinocytosis routines, and they were also the major uptake mechanisms in burn/tenotomy mice. Therefore, subsequent experiments focused on investigating the internal components of NecroMφ-EVs.

### NecroMφ-EVs Shuttled Pak4 to incur osteogenic changes of TSPCs in vitro and traumatic HO formation in vivo

**Fig. 4:**
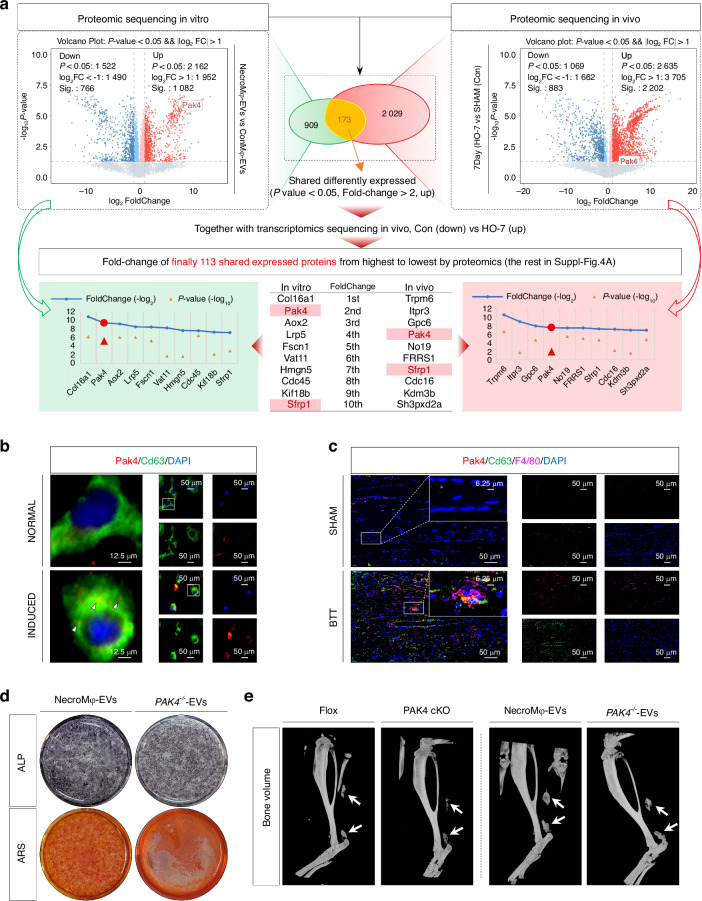
NecroMφ-EVs shuttled Pak4 to incur osteogenic changes of TSPCs in vitro and traumatic HO formation in vivo. **a** Proteomics sequencing was performed between EVs derived from PBS&DMSO stimulated BMDMs (ConMφ-EVs) and necroptotic BMDMs (NecroMφ-EVs) in vitro and between sham group and the tendon lesions at 7 days in vivo. Intersection Venn diagram was drawn, criterion: *P* value < 0.05, fold-change >2, upregulation. After further intersecting with in vivo transcriptomics sequencing between sham group and the tendon lesions at 7 days (criterion: upregulation), the fold-change of finally 113 shared expressed proteins from highest to lowest was drawn (detailed in Fig. S4A). **b** IF staining was used to detect colocalization of Pak4 (red) and Cd63 (green) in BMDMs between normal group and necroptosis induction; *n* = 6, scale bar = 50 μm (original magnification) and 12.5 μm (insert magnification of the boxed area, 4.0x). **c** IF staining was used to detect colocalization of Pak4 (red) and Cd63 (green), co-localized with F4/80 (purple), between sham group and the tendon lesions at 7 days; *n* = 5, scale bar = 50 μm (original magnification) and 6.25 μm (insert magnification of the boxed area, 8.0x). **d** ALP and ARS staining were used to detect the osteogenesis of TSPCs in addition of NecroMφ-EVs or *PAK4*^*−/−*^*-*EVs; *n* = 6. Quantification was shown in Fig. S5C. **e** Micro-CT was used to detect HO formation between the tendon lesions at 10 weeks from Flox mice and *Pak4* cKO mice, as well as in addition of NecroMφ-EVs or *PAK4*^*−/−*^*-*EVs; *n* = 5. Quantification was shown in Fig. S5F

Then, an important question that remains to be addressed was the specific content that mediated the biological effects of EVs. In vitro, proteomic sequencing was conducted to compare the EVs derived from PBS&DMSO-stimulated BMDMs (ConMφ-EVs) with those from necroptotic BMDMs (NecroMφ-EVs). Meanwhile, in vivo, proteomic sequencing was also conducted to compare control group with tendon lesions at 7 days post-injury. By integrating the results of the aforementioned in vitro and in vivo proteomics sequencing, bioinformatics analysis revealed a total of 173 differentially expressed proteins that were commonly identified in both models. The selection criteria included a *P* value of less than 0.05 and a fold change >2, together with an emphasis on upregulation. Furthermore, we intersected the aforementioned 173 proteins with the results of in vivo transcriptomics sequencing (control group vs. tendon lesions at 7 days post-injury, the same data as in Results “Macrophage necroptosis was found in tendon lesions in burn/tenotomy mice”), applying the criterion of upregulation in expression. At last, we identified a total of 113 proteins. Next, we ranked these 113 proteins based on their fold-change values in both in vivo and in vitro proteomics sequencing, ultimately identifying Pak4 and Sfrp1 as the top ten proteins in both environments. Given that Pak4 ranked higher and consistently placed within the top five across both proteomics sequencing, we finally chose to focus our subsequent investigations on Pak4 (Fig. 4a and Fig. S4A). Pak4 has been identified as a key player in fundamental cellular processes, particularly in modification and transcription regulation.^23^ This includes crucial roles in osteogenesis and stem cell differentiation.^24,25^ WB analysis (Pak4, Fig. S4B) further confirmed a higher content of Pak4 in NecroMφ-EVs compared to ConMφ-EVs, while there was a significant reduction observed in InhibNecroMφ-EVs (EVs derived from *Mlkl*^*−/−*^ BMDMs stimulated with TNFα and zVAD-fmk). Similarly, IHC staining for Pak4 (Fig. S4C) further confirmed that the expression of Pak4 was significantly elevated in burn/tenotomy mice compared to sham surgery. Additionally, a notable reduction in Pak4 expression was observed both in *Mlkl*^*−/−*^ mice and macrophage depletion mice compared to WT mice following burn/tenotomy.

To elucidate the incorporation of Pak4 into NecroMφ-EVs, IF staining was conducted to detect Pak4 co-localization with Cd63 or Eea1, markers for the endosomal system, in BMDMs subjected to necroptosis induction or not. The results demonstrated that upon necroptosis induction, an increased amount of Pak4 in the cytoplasm of BMDMs co-localized with the speckle-like distribution of Cd63 and Eea1 (Fig. 4b and Fig. S4E). This finding indicates that Pak4 is loaded into the endosomal system for EVs formation and extracellular release in response to necroptosis induction. To validate these findings in vivo, IF staining was performed on tendon tissues collected 7 days post-injury to assess co-localization of Pak4 with F4/80 and Cd63 (Fig. 4c). Compared to the sham group, injured tendons exhibited a significant elevation in Pak4 expression along with increased extracellular distribution; this change was accompanied by marked infiltration of F4/80^+^ macrophages. In terms of histological localization within the BTT group, Pak4 colocalized with Cd63, and such colocalization was found to be situated around the concentrated areas of F4/80^+^ macrophages. This suggested that these macrophages were responsible for releasing vesicles containing Pak4. Collectively, our findings indicated that necroptotic macrophages had the capacity to secrete EVs carrying Pak4. The processes involved in generating and packaging these Pak4-containing EVs were summarized in Fig. S4D.

Subsequently, to more accurately ascertain the role of Pak4 in EVs, we prepared Flox mice and *Lyz2-cre::Pak4*^*flox/flox*^ mice (conditional knockout) alongside isolated corresponding BMDMs. After the induction of necroptosis in both Flox and *Pak4* cKO BMDMs, NecroMφ-EVs and *PAK4*^*−/−*^*-*EVs were respectively isolated. Subsequently, these two types of EVs were individually added into osteo-inductive medium in vitro or administered via local tendon injection in BTT model in vivo. In vitro analysis revealed that, compared to NecroMφ-EVs, *PAK4*^*−/−*^*-*EVs significantly disabled the osteogenic behavior of TSPCs. This was evidenced through WB analysis for osteogenic markers Runx2, Ocn, and Opn (Fig. S5A), IF staining for these same markers (Fig. S5B), as well as ALP and ARS staining (Fig. 4d and Fig. S5C). Furthermore, PCR results showed that there were no significant differences among groups for *Pak4* transcription in TSPCs after PBS, NecroMφ-EVs, or *PAK4*^*−/−*^*-*EVs were added, respectively (Fig. S5D). What’s more, WB analysis showed that addition of NecroMφ-EVs led to higher Pak4 protein concentration in TSPCs than either addition of PBS or *PAK4*^*−/−*^*-*EVs, while there was no notable difference between the latter groups (Fig. S5E). The combined evidence from above demonstrated that the Pak4 protein within NecroMφ-EVs exerted an exogenous significant influence on the osteogenic behavior of TSPCs after its entry, without affecting the transcription levels of *Pak4* in TSPCs themselves. In vivo studies demonstrated that *PAK4*^*−/−*^*-*EV injection led to a reduction in mature HO and associated osteogenic indicators compared to NecroMφ-EVs injection. These findings were confirmed through IF staining for Runx2 (Fig. S5G), SOFG staining (Fig. S5H), micro-CT (Fig. 4e and Fig. S5F), and H&E staining (Fig. S5I). Moreover, comparisons between Flox mice and PAK4 cKO groups indicated a similar reduction in mature HO and osteogenic markers following the conditional knockout of *Pak4* in macrophages (Fig. 4e and Fig. S5F–I). Based on these results, it could be concluded that necroptotic macrophages secreted Pak4-enriched EVs, which were directly uptaken by TSPCs and played a crucial role in promoting the osteogenic process during the formation of HO.

### Fatty acid β-oxidation (FAO) was responsible for the biological effects of Pak4 from NecroMφ-EVs on the osteogenic behavior of TSPCs in vitro and traumatic HO formation in vivo

**Fig. 5:**
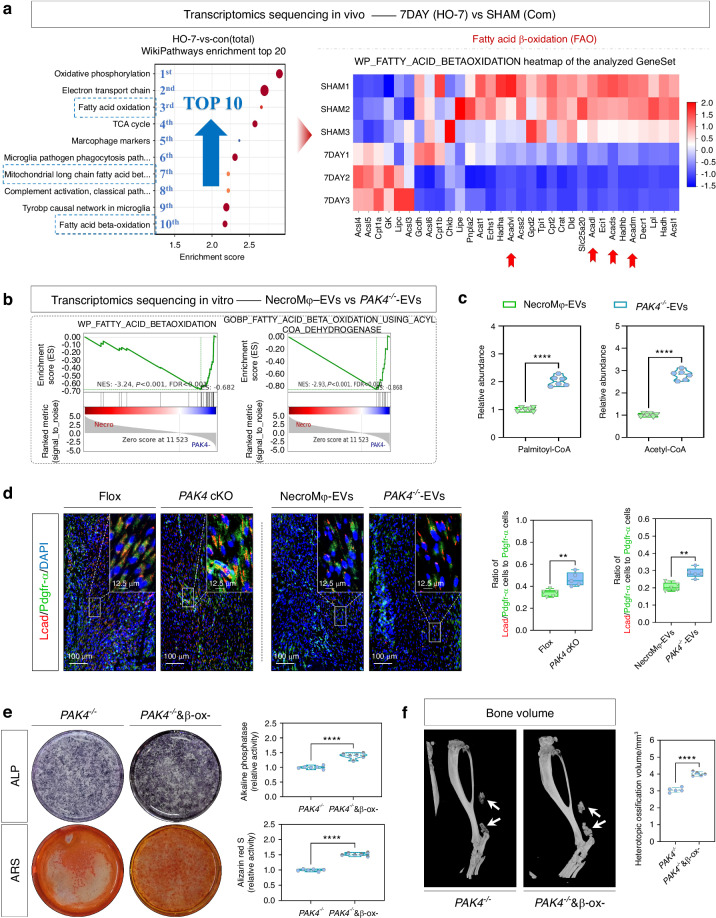
Fatty acid β-oxidation (FAO) was responsible for the biological effects of Pak4 from NecroMφ-EVs on the osteogenic behavior of TSPCs in vitro and traumatic HO formation in vivo. **a** High-throughput whole-transcriptome sequencing was performed and showed by Wikipathways enrichment (top 10) and heatmap (FAO), between the sham group and tendon lesions at 7 days; *n* = 3. **b** High-throughput whole-transcriptome sequencing was performed and showed by GSEA for “fatty acid beta-oxidation” and “fatty acid beta-oxidation using acyl-CoA dehydrogenases” between the osteogenic induced TSPCs, in addition of NecroMφ-EVs or *PAK4*^*−/−*^*-*EVs in vitro. **c** Metabolites analyses for palmitoyl-CoA and acetyl-CoA were performed to detect the FAO in osteogenic induced TSPCs, in addition of NecroMφ-EVs or *PAK4*^*−/−*^*-*EVs; *n* = 6, ****P < 0.000 1. **d** IF staining was used to detect the positive cells of Lcad (red), co-localized with Pdgfr-α (green), between the tendon lesions at 7 days from Flox mice and *Pak4* cKO mice, as well as in addition of NecroMφ-EVs or *PAK4*^*−/−*^*-*EVs; *n* = 5, **P < 0.01, scale bar = 100 μm (original magnification) and 12.5 μm (insert magnification of the boxed area, 4.0x). **e** ALP and ARS staining were used to detect the osteogenesis of TSPCs in addition of *PAK4*^*−/−*^*-*EVs, with or without *sh-Lcad*; *n* = 6, ****P < 0.000 1. **f** Micro-CT was used to detect HO formation in the tendon lesions at 10 weeks in addition of *PAK4*^*−/−*^*-*EVs, with or without *sh-Lcad*; the volume of HO was quantified from each group; *n*= 5, ****P < 0.000 1

**Fig. 6:**
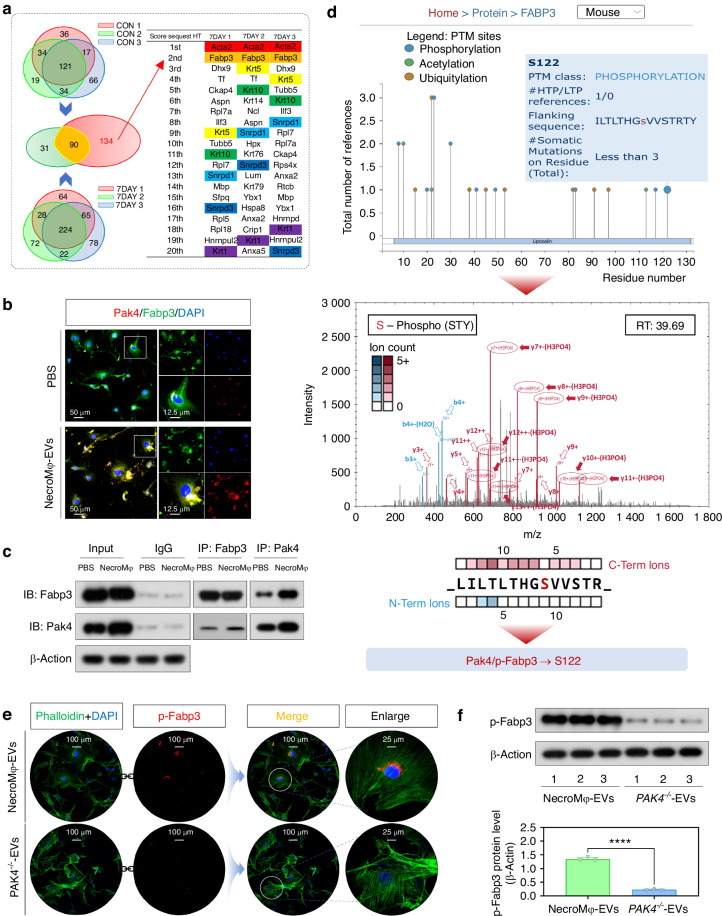
Pak4 from NecroMφ-EVs directly binds to Fabp3 at S122 phosphorylation site in TSPCs in burn/tenotomy mice. **a** Mass spectrometry analysis was used to detect the potential molecules that could bind with Pak4 between the sham group and tendon lesions at 7 days; *n* = 3. Intersection Venn diagram was drawn, criterion: identified in all three biological samples. The top 20 Score Sequest HT of finally 134 molecules (capable of directly binding to Pak4 in the BTT group but not in the control group) in 3 individual BTT group from highest to lowest was drawn. **b** IF staining was used to detect the colocalization by the positive cells of Pak4 (red) and Fabp3 (green), co-stained with DAPI (blue), in the osteogenic induced TSPCs, in addition of PBS or NecroMφ-EVs; *n* = 6, scale bar = 50 μm (original magnification) and 12.5 μm (insert magnification of the boxed area, 4.0x). **c** Co-IP analysis was used to verified the bind relationship between Pak4 and Fabp3 in the osteogenic induced TSPCs, in addition of PBS or NecroMφ-EVs; *n* = 3. **d** Phosphorylation prediction was used to predict the murine phosphorylation site of Pak4 for Fabp3. Mass spectrometry analysis was used to detect the potential phosphorylation site of Pak4 for Fabp3 in the osteogenic induced TSPCs in addition of NecroMφ-EVs or *PAK4*^*−/−*^*-*EVs. **e** IF staining was used to detect the expression of p-Fabp3 (red), co-stained with phalloidin (green) and DAPI (blue) in the osteogenic induced TSPCs, in addition of NecroMφ-EVs or *PAK4*^*−/−*^*-*EVs; *n* = 6, scale bar = 100 μm (original magnification) and 25 μm (insert magnification of the boxed area, 4.0x). **f** WB analysis was used to detect the levels of p-Fabp3 in the osteogenic induced TSPCs in addition of NecroMφ-EVs or *PAK4*^*−/−*^-EVs; *n* = 3, ****P < 0.000 1

To gain deeper insights into the influence of Pak4 derived from NecroMφ-EVs on the osteogenic behavior of TSPCs, we conducted the in vivo transcriptome sequencing (control group vs. tendon lesions at 7 days post-injury, the same data as in Results “Macrophage necroptosis was found in tendon lesions in burn/tenotomy mice”), and searched for the meaningfully enriched item (Fig. 5a) in the disease model. In the top 10 Wikipathways Enrichments, there were 3 FAO-related items including “fatty acid oxidation” at 3st, “mitochondrial long-chain fatty acid beta oxidation” at 7st, and “fatty acid beta oxidation” at 10st. In addition, the FAO also presented in the top 10 chord diagram (Fig. S6A). The transcription of acyl-CoA dehydrogenases in FAO (*Acadvl, Acadl, Acadm, and Acads*; illustration diagram in Fig. 6S6B) was significantly reduced, as demonstrated in the volcano plot and heatmap (Fig. 5a and Fig. S6A). What’s more, the results indicated that oxidative phosphorylation and electron transport chain were also accompanied by a reduction. All these demonstrates a significant reduction in FAO after burn/tenotomy model in the early stage. Further, high-throughput whole transcriptome sequencing was conducted again in vitro on TSPCs stimulated with osteo-inductive medium, which was supplemented separately with NecroMφ-EVs or *PAK4*^*−/−*^*-*EVs. GSEA revealed that both the “fatty acid beta-oxidation” and the “fatty acid beta-oxidation using acyl-CoA dehydrogenases” were significantly reduced after NecroMφ-EVs addition, compared to *PAK4*^*−/−*^*-*EVs addition (Fig. 5b), as well as the “oxidative phosphorylation” and the “electron transport chain” (Fig. S6C). These findings were accordance with the above in vivo results, and were further supported in vitro by metabolites analyses for palmitoyl-CoA and acetyl-CoA (Fig. 5c), seahorse OCR (Fig. S6D), and IF staining (Fig. S6E) for Lcad and Mcad, the important rate-limiting enzymes during FAO. Similarly, in vivo burn/tenotomy models separately received local tendon injection of NecroMφ-EVs and *PAK4*^*−/−*^*-*EVs. The results demonstrated that IF staining (Lcad and Mcad co-localized with Pdgfr-α, a marker for identifying TSPCs; Fig. 5d and Fig. S6G) and WB analysis (Lcad and Mcad, Fig. S6F) corroborated an enhanced FAO in TSPCs following the injection of *PAK4*^*−/−*^*-*EVs compared to NecroMφ-EVs. Furthermore, the in vivo results also indicated increased FAO in TSPCs associated with *Pak4* conditional knockout in macrophages when compared to the wild-type group.

Then, the effects of FAO on the osteogenic behavior of TSPCs and the traumatic HO were evaluated. AVs in vitro and AAVs in vivo designed to downregulate *Lcad* were employed to inhibit FAO (Fig. S7A). In vitro, TSPCs were stimulated with an osteo-inductive medium supplemented with *PAK4*^*−/−*^*-*EVs. Compared to the sh-NC control group, the osteogenic behavior of TSPCs was significantly enhanced upon the addition of *sh-Lcad*, as demonstrated by WB analysis (Runx2, Ocn, and Opn; Fig. S7B), IF staining (Runx2, Ocn, and Opn; Fig. S7C), as well as ALP and ARS staining (Fig. 5e). In vivo, in a burn/tenotomy model involving local tendon injection of PAK4^−/−^-EVs, compared to the sh-NC control group, there was a significant increase in mature HO and osteogenic indicators when *sh-Lcad* was injected. This finding was confirmed through IF staining (Runx2; Fig. S7D), SOFG staining (Fig. S7E), micro-CT (Fig. 5f), and H&E staining (Fig. S7F). These results suggested that Pak4 from NecroMφ-EVs enhanced the osteogenic behavior of TSPCs during traumatic HO formation by reducing FAO.

### Pak4 from NecroMφ-EVs reduced FAO of TSPCs in burn/tenotomy mice by directly binding to Fabp3

To further decipher how Pak4 influenced FAO in TSPCs, we investigated the downstream binding molecules associated with EVs-shuttled Pak4. Mass spectrometry analysis was conducted to compare the control group and tendon lesions at 7 days, aiming to identify potential molecules capable of binding with Pak4 in the disease model (Fig. 6a and Fig. S8A). Results indicated that, following the intersection of results from three biological samples in both the control group and the BTT (7 days post-injury) group, there were 121 molecules identified in the control group and 224 molecules in the BTT group capable of directly binding to Pak4, respectively. We further conducted an intersection analysis of the molecules identified in the control group (121) and those in the BTT group (224). Ultimately, we identified 134 molecules that were capable of directly binding to Pak4 in the BTT group but not in the control group. Based on the capacity for direct binding with Pak4, Acta2, Fabp3, and Krt5 were ranked among the top 10 molecules across all three BTT samples. We chose FABP3 for subsequent research, considering it is also a general fatty acid metabolism-related molecule. Consistently, molecular docking (Fig. S8B) validated the direct physical binding ability between Pak4 and Fabp3. In vitro study, IF staining for Pak4 and Fabp3 (Fig. 6b) further visualized this direct interaction, revealing a higher degree of colocalization in TSPCs treated with NecroMφ-EVs compared to those receiving PBS. Co-IP (Fig. 6c) further confirmed the physical association between Pak4 and Fabp3. In vivo study, IF staining demonstrating colocalization of Pak4 and Fabp3 with Pdgfr-α (Fig. S8C) revealed increased colocalization in BTT mice compared to the sham group. Co-IP experiments also provided support for the physical association between Pak4 and Fabp3 (Fig. S8D). Collectively, these results indicated that Pak4 directly interacted with Fabp3 in TSPCs exhibiting osteogenic behavior and the disease model of trauma-induced HO.

**Fig. 7:**
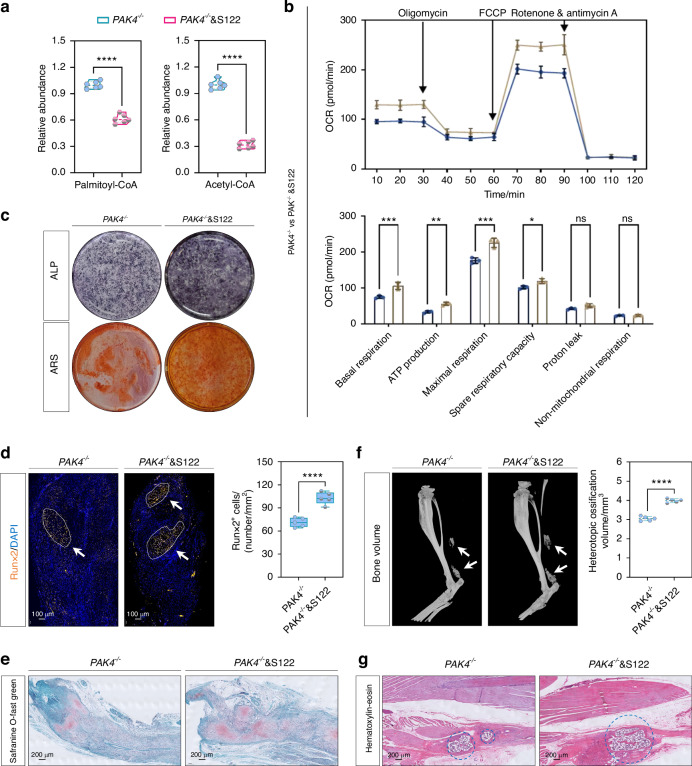
Phosphomimetic mutant of Fabp3 on S122 site reduced FAO of TSPCs and increased traumatic HO formation in burn/tenotomy mice. **a** Metabolites analyses for palmitoyl-CoA and acetyl-CoA were performed to detect the FAO in osteogenic induced TSPCs, in addition of *PAK4*^*−/−*^*-*EVs, with or without *Fabp3*^*S122D*^; *n* = 6, ****P < 0.000 1. **b** Seahorse test was used to detect the oxidative phosphorylation level in the osteogenic induced TSPCs in addition of *PAK4*^*−/−*^*-*EVs, with or without *Fabp3*^*S122D*^; *n* = 3,  represented PAK4^-/-^& S122 group, and  represented PAK4^−/−^ group, *P < 0.05, **P < 0.01, ***P < 0.001, ns, no significant difference. **c** ALP and ARS staining were used to detect the osteogenesis of TSPCs in addition of *PAK4*^*−/−*^*-*EVs, with or without *Fabp3*^*S122D*^; *n* = 6. **d** IF staining was used to detect osteogenesis by the positive cells of Runx2 (orange) in the tendon lesions at 3 weeks in addition of *PAK4*^*−/−*^*-*EVs, with or without *Fabp3*^*S122D*^; *n* = 5, scale bar = 100 μm (original magnification), ****P < 0.000 1. **e** SOFG staining was used to detect osteogenesis region in the tendon lesions at 3 weeks in addition of *PAK4*^*−/−*^*-*EVs, with or without *Fabp3*^*S122D*^; *n* = 5, scale bar = 200 μm (original magnification). **f** Micro-CT was used to detect HO formation in the tendon lesions at 10 weeks in addition of *PAK4*^*−/−*^*-*EVs, with or without *Fabp3*^*S122D*^; the volume of HO was quantified from each group; *n* = 5, ****P < 0.000 1. **g** HE staining was used detect ossification region in the tendon lesions at 10 weeks, in addition of *PAK4*^*−/−*^*-*EVs, with or without *Fabp3*^*S122D*^; *n* = 5, scale bar = 200 μm (original magnification)

Due to Pak4 being a type of kinase for serine/threonine proteins, phosphorylation prediction and mass spectrometry analyses were conducted to identify the potential phosphorylation site of Pak4 on Fabp3. Both approaches pointed to S122 as the target (Fig. 6d). Subsequently, we assessed changes in the phosphorylation level of Fabp3 (p-Fabp3) upon knockout of *Pak4*. IF staining (p-Fabp3, Fig. 6e and Fig. S8E) and WB analysis (p-Fabp3, Fig. 6f and Fig. S8F) further validated that p-Fabp3 levels were reduced following *Pak4* knockout, both in vitro and in vivo. To confirm that direct phosphorylation of Fabp3 at the S122 site constitutes a downstream pathway through which Pak4 from NecroMφ-EVs affects FAO in TSPCs, we engineered a phosphomimetic mutant of Fabp3 on S122 site called *Fabp3*^*S122D*^. In vitro experiments demonstrated that transfection with the plasmid encoding *Fabp3*^*S122D*^ led to decreased FAO, as indicated by metabolite analyses revealing lower levels of palmitoyl-CoA and acetyl-CoA (Fig. 7a), alongside seahorse OCR supporting these findings (Fig. 7b). Furthermore, enhanced osteogenic activity was observed through WB analysis measuring Runx2, Ocn, and Opn expression levels (Fig. S9A), IF staining for osteogenic markers Runx2, Ocn, and Opn (Fig. S9B), as well as ALP and ARS staining (Fig. 7c). Consistently, in vivo assessments after transfecting AAV encoding *Fabp3*^*S122D*^ revealed an increase in mature HO and osteogenic indicators, as evidenced by IF staining for Runx2 (Fig. 7d), SOFG staining (Fig. 7e), micro-CT (Fig. 7f), along with H&E staining (Fig. 7g). Collectively, these results indicated that Pak4 derived from NecroMφ-EVs reduced FAO in TSPCs by directly phosphorylating downstream target Fabp3 at the S122 site, ultimately incurring osteogenic changes of TSPCs during traumatic HO formation.

### PAK4 and FABP3-related FAO were found to be in an activated state in the human HO tissues

**Fig. 8:**
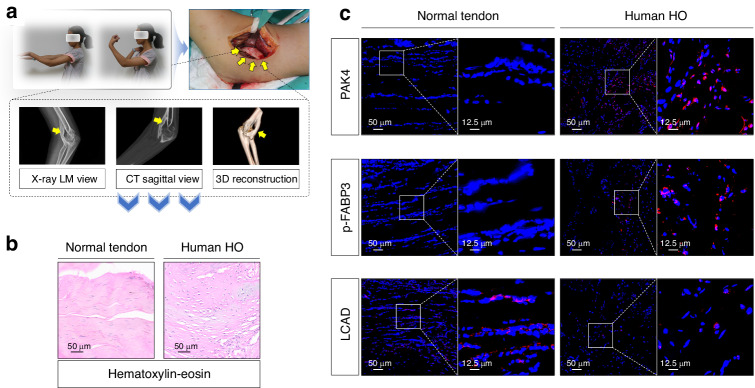
PAK4 and FABP3-related FAO were found to be in an activated state in the human HO tissues. **a** Schematic depiction of human traumatic HO tissues. **b** H&E staining was used to detect ossification region between HO tissue section and normal tendon tissue section; *n* = 5, scale bar = 50 μm (original magnification). **c** IF staining was used to detect the positive cells of PAK4 (red), p-FABP3 (red), and LCAD (red) between HO tissue section and normal tendon tissue section; *n* = 5, scale bar = 50 μm (original magnification)

Finally, the activation of PAK4, p-FABP3, and LCAD was examined in clinical samples to establish the clinical relevance of our findings. Soft tissue-derived HO samples and control soft tissue samples (normal tendon) were collected (Fig. 8a). H&E staining revealed their characteristic histological features, which allowed for the identification and definition of the ossification region in HO samples (Fig. 8b). Consequently, stronger IF staining for PAK4 and p-FABP3, as well as reduced levels of LCAD, were observed in HO tissues compared to normal tendon tissues (Fig. 8c). This expression pattern paralleled that seen in murine models. Collectively, these data suggested that cells within human HO tissues were also activated for PAK4 and FABP3-related FAO, indicating a trend similar to that observed in murine tissue sections.

## Discussion

**Fig. 9:**
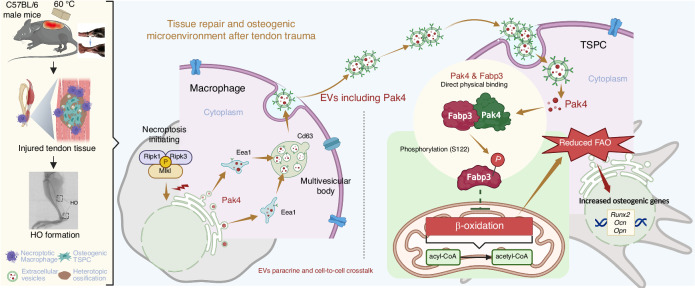
Graphical summary. Following soft tissue injury, macrophages infiltrate, and necroptosis occurs. Concurrently, this process is accompanied by paracrine EVs-derived PAK4, which subsequently regulates FABP3 in TSPCs and reduces FAO. This cascade ultimately leads to osteogenic behavior in TSPCs and contributes to HO formation. Notably, PAK4 binds directly to FABP3 and phosphorylates it at the S122 site, thereby influencing FAO

The complex pathological nature of traumatic HO has attracted considerable attention, particularly concerning the critical role of the crosstalk between immune cells and TSPCs in their osteogenic behavior. To the best of our knowledge, this study represents the first elucidation of how PAK4, secreted by necroptotic macrophages, reduces FABP3-mediated FAO and enhances osteogenic activity in TSPCs during traumatic HO formation (mechanism diagram in Fig. 9). Our findings offer novel insights into potential therapeutic strategies for managing traumatic HO as well as other diseases associated with necroptosis and fatty acid metabolism.

Macrophages are critical components of the peripheral immune system, playing a pivotal role in regulating local tissue homeostasis through the secretion of paracrine inflammatory factors and cytokines.^26^ The microenvironment’s homeostasis significantly influences the quality of tissue repair. For instance, macrophages have been identified as key players in the regulation of fracture healing by orchestrating immune responses, facilitating angiogenesis, and interacting with other cell types; this underscores their importance in tissue repair processes.^27^ To date, most literature highlights the close relationship between macrophages and HO formation; however, there is limited research on the status and morphological changes that occur within macrophages during HO development.^5^ Therefore, what specific pathological alterations occurred in macrophages during HO formation? In this study, GSEA and bioinformatics analysis reveal an increased enrichment of cellular responses to tumor necrosis factor as well as necroptotic signaling pathways in the burn/tenotomy model compared to the sham group. Furthermore, macrophage necroptosis is confirmed during the early stages of HO formation. After trauma and injury, various stimulating factors, including damage-associated molecular patterns (DAMPs) and pathogen-associated molecular patterns (PAMPs), are widely present. These factors contribute to increased oxidative stress responses and enhanced production of mitochondrial reactive oxygen species (mtROS) in macrophages, ultimately triggering necroptosis.^28^ Necroptotic macrophages have been demonstrated to play a significant role in a wide range of acute injuries and inflammation-related diseases, such as acute liver injury,^29^ acute myocardial infarction,^30^ and acute asthma.^31^ This involvement occurs through cell-to-cell component transport within the microenvironment.^17,32^ Macrophages can rapidly migrate into damaged tissues at the initial phase of tissue repair and initiate crosstalk that directly influences the lineage differentiation direction of various surrounding stem cells.^33,34^ Normally, after tendon injury, TSPCs are enriched during the early phase of tendon healing, followed by tenogenic differentiation in the later phase, which can lead to improved outcomes in tendon healing.^35^ However, under the influence of a local osteogenic microenvironment following trauma, TSPCs exhibit abnormal differentiation and begin to shift towards osteogenesis. Our previous studies have demonstrated that tendon cells marked by PDGFRα in human HO tissues exhibit a pronounced tendency towards osteogenic differentiation, indicating that HO formation results from abnormal repair processes and is influenced by the fate change in TSPCs differentiation.^20^ Therefore, are necroptotic macrophages involved in HO formation by affecting osteogenic behavior of TSPCs? In this study, we observed an increase in the osteogenic potential of TSPCs when co-cultured with necroptotic BMDMs. Moreover, both the ability of osteogenesis in TSPCs and HO formation were reduced when using *Mlkl*^*−/−*^ mice to eliminate necroptosis. These findings suggest that macrophage necroptosis contributes significantly to the osteogenic differentiation of TSPCs during HO formation.

How do necroptotic macrophages induce the osteogenic differentiation of TSPCs during HO formation? Paracrine signaling represents the most critical form of communication between cells within local tissues. Previous studies have reported that macrophages secrete a variety of soluble factors and extracellular vesicles, including oncostatin M, prostaglandin E2, and bone morphogenetic protein-2, which collectively regulate stem cell differentiation and promote tissue repair as well as bone healing.^36,37^ Therefore, which paracrine component mediates the effect of necroptotic BMDMs on TSPCs differentiation? This study identifies NecroMφ-EVs as the primary factor involved in this process; notably, the osteogenic activity is significantly greater than that observed in the NecroMφ-SFs group both in vitro and in vivo. EVs derived from various cell types play a crucial role in the osteogenic differentiation of stem cells, serving as a communication bridge between cells. In addition to macrophages, which have been extensively studied, other cell types such as neutrophils, nerve cells, and muscle cells also release EVs that participate in the regulation of osteogenesis.^38–40^ Notably, there is one published article addressing the intercellular crosstalk involving EVs during the formation of neurogenic HO. In Lu et al.‘s study, the authors demonstrate that traumatic brain injury prompts the release of brain-derived extracellular vesicles (BEVs), which contain pathogens and facilitate inter-organ communication. Within injured tendons, phagocytosis of BEVs by fibroblasts triggers pyroptosis, resulting in elevated concentrations of calcium and phosphorus and creating a microenvironment conducive to osteogenesis.^39^ Then, which molecule mediates necroptotic macrophages’ paracrine effects on TSPCs? This study demonstrates that Pak4 is among the top five proteins identified in both in vitro and in vivo proteomics sequencing. In a previous study, the Pak4 pathway was found to play a crucial role in maintaining osteogenesis homeostasis. Immunoprecipitation assays confirmed a direct interaction between Pak4 and β-Catenin, which is recognized as a key factor in osteogenic differentiation.^24^ Therefore, can Pak4 from NecroMφ-EVs incur osteogenic changes of TSPCs? In this study, the knockout of *Pak4* significantly impaired the osteogenic behavior of TSPCs and reduced ectopic bone formation in the burn/tenotomy model. These findings indicate that necroptotic macrophages secrete Pak4, contributing to the osteogenic differentiation of TSPCs during HO formation.

In the local microenvironment following injury, alterations in energy metabolism play a crucial role in modulating the biological behavior and function of stem cells. The fundamental processes involved in energy metabolism encompass glycolysis within the cytoplasm, the tricarboxylic acid cycle (TCA) and FAO occurring in mitochondria, and others.^41^ During normal tissue repair, stem cell metabolism achieves normal lineage differentiation in a homeostasis. However, perturbations in the microenvironment lead to energy metabolic reprogramming of stem cells, which consequently results in aberrant differentiation.^42^ For instance, Wang et al. demonstrate that muscle injury promotes fatty acid uptake in muscle stem cells, subsequently enhancing FAO to facilitate their proliferation and support muscle regeneration. Furthermore, the restriction of FAO impairs muscle regeneration in mice, underscoring the vital role of FAO in the process of muscle repair.^43^ Therefore, is there any change in energy metabolism pattern in TSPCs influenced by Pak4? In this study, both Wikipathway and GSEA enrichment analyses demonstrate that the gene sets associated with FAO exhibit significant alterations in BTT mice compared to sham surgery. Furthermore, these changes are also observed following *Pak4* knockout, both in vitro and in vivo. These findings indicate that FAO is modified during the osteogenic differentiation of TSPCs. Mitochondrial FAO represents the primary metabolic pathway for the catabolism of fatty acids and is essential for sustaining energy balance within the human body. Fatty acids act as a crucial energy source during periods of fasting and in post-absorptive states when glucose availability is limited. Furthermore, even in conditions where glucose is abundantly available, FAO continues to serve as a significant energy source for vital organs such as the heart, skeletal muscle, and kidneys. Mitochondrial FAO is responsible for the degradation of fatty acids into acetyl-CoA, subsequently contributing to adenosine triphosphate (ATP) synthesis by providing substrates for the TCA and oxidative phosphorylation. The status of fatty acid metabolism plays a pivotal role in regulating stem cell differentiation. Carnitine palmitoyl transferase 1 (CPT1), a key enzyme in FAO, plays a significant role in enhancing the reprogramming efficiency of pluripotent stem cells by upregulating oxidative phosphorylation and downregulating protein kinase C activity.^44^ Furthermore, CPT1 is instrumental in maintaining neural stem/progenitor cells and ensuring proper neurogenesis through the modulation of malonyl-CoA levels.^45^ In this study, after using AAV and AV downregulating *Lcad* as FAO inhibitor, results show increased osteogenic behavior of TSPCs in vitro and ectopic bone in vivo. These results indicate that FAO is responsible for the promoted effects of Pak4 from NecroMφ-EVs on the osteogenic behavior of TSPCs during traumatic HO formation.

What is the downstream mechanism by which Pak4 regulates FAO in TSPCs? In this study, series experiments including mass spectrometry, molecular docking, IF staining for colocalization, and co-IP find that Pak4 directly bound to Fabp3. FABPs are intracellular proteins that play a key role in FAO by transporting and regulating fatty acids, influencing energy metabolism and cell function. FABPs transport fatty acids from cell membranes to mitochondria and peroxisomes, ensuring a supply of FAO substrates.^46^ To date, researchers have identified nine distinct isoforms of this protein, all sharing the mechanism of interaction with lipid ligands to facilitate their transfer to metabolic sites. Among these isoforms, FABP3 is expressed at the highest levels in cardiac and skeletal muscle tissues. As a lipid chaperone, FABP3 is essential for maintaining homeostasis in intracellular energy metabolism and plays an important role in the repair processes associated with acute heart and tendon injuries.^47,48^ Furthermore, FABP3 influences the differentiation of stem cells also by regulating cellular autophagy, which promotes adipogenesis while inhibiting osteogenesis in mesenchymal stem cells (MSCs). Notably, the knockdown of *Fabp3* has been shown to alleviate bone loss.^49^ Therefore, is Fabp3 involved in Pak4 mediated FAO in TSPCs? In this study, mass spectrometry confirms S122 as the phosphorylation site of Pak4 acting on Fabp3, and the osteogenic behavior of TSPCs and ectopic bone in burn/tenotomy mice is increased after using *Fabp3*^*S122D*^ for a phosphomimetic mutant. These results indicate that Fabp3 is the vital downstream pathway for fatty acid metabolism in TSPCs mediated by Pak4. Similarly, as a kinase, Pak4 phosphorylates Ncor1 at T1619/T2124 in non-alcoholic fatty liver disease, resulting in an increase in its nuclear localization and interaction with Ppar-α, thereby regulating FAO.^50^

There are several limitations and prospects for this research. Firstly, from Fig. 3, both EVs and SFs significantly contribute to the osteogenic behavior of TSPCs in vitro and traumatic HO formation in vivo; however, we do not study on SFs in this study as the effects of EVs are greater. Indeed, soluble molecules in SFs can also be promising topic in future study, which could undergo downstream cascade reactions by such as binding to receptors on the surface of the target cell membrane.^51,52^ Secondly, this study provides evidence that macrophage necroptosis reduces FAO in TSPCs through paracrine EVs-derived Pak4, consequently increasing the osteogenic differentiation capacity of TSPCs and the formation of HO, including how NecroMφ-EVs-derived Pak4 regulates FAO in detail and the correlation of FAO with osteogenesis and HO in TSPCs. However, we do not focus on the detail of how reduced FAO increases osteogenesis and HO in TSPCs. This would be another interesting topic, while is beyond the scope of this research and can be explored in future. Combining with the published literatures, we have several hypotheses: (1) reduced FAO leads to decreased oxidative phosphorylation and electron transport chain, that may elevate the production of mitochondrial reactive oxygen species,^53,54^ which has been proved to participate in increased TSPCs osteogenesis and HO formation in our previous study^55^; (2) decreased FAO accompanies by reduced palmitoyl-CoA, that may reduce intracellular palmitoylation modification, which is reported to effectively increase the osteogenic differentiation ability.^56^ Thirdly, while our study highlighted the role of paracrine EVs-derived Pak4 in regulating Fabp3 and reducing FAO to promote HO formation, it is likely that other mechanisms can also lead to HO by decreasing FAO. For instance, inhibiting key enzymes like CPT1, which limits fatty acid transport into mitochondria for oxidation, can effectively lower FAO.^57^ Hormonal factors may also be influential; insulin resistance can reduce FAO in adipocytes and muscle cells.^58^ Similar effects might occur in TSPCs. Future research will enhance our understanding of the complex regulation of FAO in HO development. Fourthly, various catabolic pathways have been reported to be associated with the differentiation of stem cells, such as for BMDMs. These pathways include glycolysis, the pentose phosphate pathway, and amino acid metabolism.^59,60^ Currently, there is limited research on the metabolism of TSPCs. However, this study only focuses exclusively on FAO. Notably, Fig. 5a illustrates that besides FAO, glycolysis and gluconeogenesis, as well as amino acid metabolism, exhibit significant alterations in the burn/tenotomy model. This observation suggests the possibility of additional metabolic mechanisms involved in HO formation that can be investigated in future studies. Fifthly, the stage of HO in human samples utilized in this study is maturation rather than the early inflammatory stage. While immature HO (in the early stage) better represents the altered mechanisms involved in the initial phases of formation. However, acquiring immature HO poses significant challenges due to ethical considerations, as maturity is a necessary criterion for performing HO resection.^61,62^ Finding a way to acquire immature HO in a legal and ethical manner would be a promising topic for future exploration. Sixthly, the development of new drugs is primarily divided into 4 stages: drug discovery, preclinical research, clinical research, and new drug application (NDA). In vitro and in vivo studies (including large animal experiments) should be conducted for preclinical research encompassing pharmacokinetics (PK), pharmacodynamics (PD), and toxicology (TOX) assessments to further validate the safety, efficacy, and optimal administration routes of the new drug, etc. Drug selection and clinical translation is a lengthy, highly significant, and exceedingly rigorous process. We hope that the findings of this study, particularly those related to PAK4, alongside necroptosis regulators such as RIPK1, RIPK3, and MLKL, as well as FAO, can provide references for the design of targets in the future clinical prevention and treatment of post-traumatic HO.

## Materials and methods

### Reagents and materials

Fetal bovine serum (FBS) and alpha-minimum essential medium (α-MEM) were purchased from Gibco (Carlsbad, CA). Penicillin/streptomycin was purchased from HyClone (Logan, UT, USA). zVAD-fmk (Cat# HY-16658B), Pitstop 2 (Cat# HY-115604), nystatin (Cat# HY-17409), and EIPA (Cat# HY-101840) were purchased from MedChemExpress (New Jersey, USA). TNFα (Cat# CF09) was purchased from Novoprotein (Suzhou, China). Adeno-associated virus (AAV) and adeno virus (AV) for downregulating long-chain acyl-CoA dehydrogenase (*sh-Lcad*), as well as AAV and plasmid for variant of S122 on Fabp3 phosphorylation site (a phosphomimetic mutant *Fabp3*^*S122D*^), along with their corresponding controls, were constructed from Genomeditech Co., Ltd (Shanghai, China). The p-Ripk1 (Cat# 28252-1-AP), Runx2 (Cat# 20700-1-AP), Opn (Cat# 22952-1-AP), Cd9 (Cat# 60232-1-lg), Cd81 (Cat# 27855-1-AP), Alix (Cat# 12422-1-AP), Tsg101 (Cat# 28283-1-AP), Calnexin (Cat# 10427-2-AP), Pak4 (Cat# 14685-1-AP), Eea1 (Cat# 28347-1-AP), Lcad (Cat# 17526-1-AP) and Fabp3 (fatty acid binding protein 3, Cat# 10676-1-AP) antibodies were purchased from Proteintech (Chicago, IL, USA). The Ocn (Cat# ab93876) antibody was purchased from Abcam (USA). The Pdgfr-α (Cat# AF1062) antibody was purchased from R&D Systems (Minneapolis, MN, USA). The p-Ripk3 (Cat# 91702), p-Mlkl (Cat# 37333), and F4/80 (Cat# 70076) antibodies were purchased from Cell Signaling Technology (CA, USA). The Mcad (Cat# A4567) antibody was purchased from Abclonal (Wuhan, China). The Tnf-α (Cat# GB115702-100) and β-Actin (Cat# GB15001) antibodies were purchased from Servicebio (Wuhan, China). The p-Fabp3 antibody was constructed from Genscript (Nanjing, China).

### Murine trauma-induced HO model (burn/tenotomy, BTT), specimen collection, and histological observations

Male C57BL/6 mice, aged 6 weeks, were bred in a special pathogen-free environment at the Animal Experimental Center of Shanghai Sixth People’s Hospital. The mice had unlimited access to food and water and were given a minimum acclimatization period of one week prior to modeling. All procedures involving animal experiments received approval from the Institutional Animal Care and Use Committee of Shanghai Sixth People’s Hospital (No: DWSY2021-0064), and were conducted in accordance with the National Institutes of Health Guidelines for the Care and Use of Laboratory Animals.

Wild‐type C57BL/6 mice (WT) were provided by the Shanghai laboratory animal center (SLAC), while the *Mlkl* (*Mlkl*^*−/−*^) general knock‐out and *Pak4* (*Lyz2-cre::Pak4*^*flox/flox*^) conditional knockout mice, as well as their control mice, were acquired from GemPharmatech (Nanjing, China, *Mlkl*^*−/−*^ | Strain NO. T002470; *Lyz2-cre::Pak4*^*flox/flox*^ | Strain NO. T018538) and housed in the specific pathogen‐free facility. Primers used for genotyping the *Mlkl*^*−/−*^ and *Lyz2-cre::Pak4*^*flox/flox*^ mice were listed in Tables S1 and S2. Here is the summary of *Mlkl* Cas9-KO Strategy. This model used CRISPR/Cas9 technology to edit the *Mlkl* gene. Generally, the *Mlkl* gene has 4 transcripts, and exon 8 of *Mlkl*-201 (ENSMUST00000056157.13) transcript is recommended as the knockout region. Knock out this region results in disruption of protein function. In this project, the brief process was as follows: sgRNA was transcribed in vitro. Cas9 and sgRNA were microinjected into the fertilized eggs of C57BL/6J mice. Fertilized eggs were transplanted to obtain positive F0 mice, which were confirmed by PCR and sequencing. A stable F1 generation mouse model was obtained by mating positive F0 generation mice with C57BL/6J mice. Detailed information can be acquired in https://en.gempharmatech.com/product/details100035_3911059.html. Similarly, information for *Pak4* cKO strategy can be found in https://en.gempharmatech.com/product/details100035_3699461.html.

In this study, we employed a murine burn/tenotomy model to simulate traumatic HO, following the methodology described by Peterson.^21^ Mice were anesthetized using 1% pentobarbital sodium, and a longitudinal incision of ~0.5 cm was made on the medial aspect of both distal hindlimbs. The Achilles tendon was exposed and transected at its midpoint, after which the incision was closed with a 5–0 Vicryl suture. Subsequently, an aluminum block measuring 2 × 2 × 3 cm and weighing 35 g, covering ~30% of the total body surface area, was preheated to 60 °C in a water bath. Following tenotomy, the aluminum block was placed on the shaved dorsal skin for a duration of 17 s. The sham surgery involved only exposing the Achilles tendon without performing either tenotomy or burned injury. For in vivo transduction and injection, all AAVs, EVs, and SFs were administered via localized tendon injection at the injury site, starting at the time of surgery and continued once a week for three weeks.^52^ For AAVs, the dosage per time for each tendon involved 1 × 10^10^ plaque-forming units in 10 μL PBS. For EVs, the dosage per time for each tendon was 20 μg in 10 μL PBS. For SFs, we used Amicon® Ultra filters (Sigma-Aldrich, US) to concentrate the extracted supernatants through two consecutive rounds of filtration. In the first round, we concentrated 15 mL of supernatants to 200 µL using Ultra-15. In the second round, we further concentrated this obtained 200 µL (0.2 mL) to 20 µL using Ultra-0.5. The dosage per time for each tendon was 10 μL from this final volume of 20 μL. Detailed process was shown in “EVs isolation and characterization and Fig. 3a.”

Mice were sacrificed at various time points to facilitate histological observation at 7 days, 3 weeks, and 10 weeks, with the aim of detecting inflammation, osteogenesis, and mature HO, respectively. Specifically, skin samples harvested from the lower extremities were meticulously excised, and soft tissue specimens were obtained from the musculotendinous junction to the calcaneus enthesis. The tissues were fixed in a 10% (v/v) formalin solution. For tissues collected at the 10-week mark, an additional decalcification step employing a 19% ethylenediaminetetraacetic acid solution was performed. Following decalcification, the tissues underwent dehydration and embedding in paraffin wax. Longitudinal sections of the Achilles tendon with a thickness of 5 μm were prepared for subsequent analysis. A deparaffinization and rehydration process was conducted on stained sections mounted on Thermo Superfrost® Plus slides in preparation for further staining procedures. High-throughput whole transcriptome sequencing, proteome sequencing, western blotting (WB), routine histological analyses (Safranine O-fast green [SOFG], haematoxylin and eosin [H&E]), immunohistochemistry (IHC), immunofluorescence (IF) staining, as well as micro-computed tomography (micro-CT), were employed to analyze changes in tissue morphology and composition.

### High-throughput whole transcriptome sequencing

The soft tissue at the Achilles tendon underwent whole transcriptome genome sequencing, provided by Shanghai OE Biotech Co., Ltd. The following steps were conducted: total RNA was extracted, ribosomal RNA was digested using a Ribo-Zero kit, and the RNA was fragmented into short segments with the addition of an interruption reagent. A cDNA chain was synthesized using the interrupted RNA as a template alongside random primers consisting of six bases. A two-strand synthesis reaction system was prepared to synthesize double-stranded cDNA. During this process, dUTP replaced dTTP in the cDNA synthesis; subsequently, one strand containing dUTP was digested using UNG enzyme method to isolate only those cDNAs corresponding to different junctions on that strand. A purification kit was employed to isolate one strand of cDNA, which was then repaired at its ends and had an a-tail added for sequencing purposes. Fragment size selection and PCR amplification were performed prior to qualifying the constructed libraries utilizing Agilent 2100 Bioanalyzer. The Illumina sequencer facilitated subsequent sequencing processes.

### Enzyme‐linked immunosorbent assay

Concentrations of the cytokine Tnf-α in tissue homogenates were measured using ELISA kits (Anogen, Canada) according to the manufacturer’s instructions. The total protein content of the samples was determined with a BCA protein assay kit (Beyotime, China).

### Western blotting

Tissues (in vivo) or whole cells (in vitro) from different groups were collected and lysed using radioimmunoprecipitation assay (RIPA) lysis buffer, supplemented with a protease inhibitor cocktail (Servicebio, Wuhan, China). The lysate was sonicated on ice, and total tissue protein was extracted by centrifugation at 12 000 r/min for 10 min. Protein concentrations were determined using a bicinchoninic acid (BCA) protein detection kit (Servicebio, Wuhan, China). The protein samples (30 μg each) were mixed with sodium dodecyl sulfate‐polyacrylamide gel electrophoresis loading buffer (SDS‐PAGE) (Servicebio, Wuhan, China), followed by heat treatment at 100 °C for 5 min. Subsequently, the proteins were separated via electrophoresis on a 12% SDS‐PAGE gel. Afterward, the separated proteins were transferred onto a polyvinylidene difluoride membrane. Following blocking of the membrane with 5% non-fat milk or bovine serum albumin for 1 h at room temperature, it was incubated overnight at 4 °C with specific primary antibodies against p-Ripk1, p-Ripk3, p-Mlkl, Pak4, Lcad, Mcad, p-Fabp3, Fabp3, Runx2, Ocn, Opn, and β-Actin. After this incubation step, HRP-conjugated secondary antibodies were applied to the membrane for an hour at room temperature. Finally, a chemiluminescence reagent was utilized to visualize signals detected using a ChemiDoc CRS imaging system (Bio-Rad, USA). The relative density of each gray protein band was quantified utilizing ImageJ software (version 1.32, National Institutes of Health). In brief, the integrated optical density (IntDen) of each target protein band was measured, whereupon normalization allowed calculation of the relative gray level in comparison to that of the internal reference protein β-Actin.

### Safranine O-fast green staining

The tissue sections were dewaxed using xylene, followed by dehydration with a gradient alcohol solution. Subsequently, the sections underwent Hematoxylin drop staining for 5 min, after which they were rinsed with tap water to eliminate excess dye and treated with an acid differentiation solution for an additional 15 s. The sections were then immersed in distilled water for another 10 min. Solid green dye drop staining was applied for 5 min, during which any excess dye was gently shaken off without subsequent washing. A weak acid solution was utilized to wash the film for 15 s in order to remove any remaining solid green dye, and the excess liquid was eliminated by shaking without further washing. Next, the samples underwent Saffron O drop staining for 5 min, again removing any excess dye without washing. Following this procedure, gradual dehydration of the samples was achieved using a gradient ethanol solution until transparency was obtained, employing xylene as a final clearing agent. Finally, the specimens were sealed with resin after being rendered transparent with xylene prior to observation and photography.

### Haematoxylin and eosin staining

The dewaxing process commenced with the application of xylene, followed by hydration through a gradient of alcohol concentrations to enhance dye penetration into the tissue. Subsequently, hematoxylin dye was employed for staining, after which it was gently rinsed with running water and differentiated using a 0.1% ethanol hydrochloride solution. Excess dye was meticulously removed through thorough washing with water prior to eosin staining. After eosin staining, any remaining excess dye was again washed away with running water, followed by dehydration utilizing a gradient of alcohol concentrations. Finally, tissue samples were made transparent using xylene and subsequently sealed with resin for further observation and photography.

### Immunohistochemistry and immunofluorescence staining

The sections were prepared, and subsequently, a citrate buffer was employed for thermal remediation to retrieve antigens. Following this step, the sections were inactivated using hydrogen peroxide and blocked with goat serum. The next stage involved incubating the sections overnight with primary antibodies targeting p-Ripk1, p-Ripk3, p-Mlkl, Pak4, Lcad, Mcad, p-Fabp3, and Fabp3. On the following day, for immunohistochemical (IHC) staining, the sections were treated with Horseradish Peroxidase (HRP)-conjugated secondary antibodies. For immunofluorescence (IF) staining, sections underwent a 1-h incubation at 37 °C with either Alexa Fluor 488-conjugated secondary antibodies or Cy3-matched species. Nuclear counterstaining was conducted using 4,6-diamidino-2-phenylindole (DAPI; Cat #P0131; Beyotime Institute of Biotechnology). The field of interest for this study is shown in Fig. S10.

### Microcomputed tomography scanning

At the end of the 10-week period, euthanasia was conducted on the mice, followed by the harvesting of their hindlimbs. These specimens were then preserved in a formalin solution (10%, v/v) for a duration of 48 h. Subsequently, the fixed hindlimbs underwent imaging using a Skyscan 1176 high-resolution micro-CT scanner (software version 1.1 [build 6], Bruker) at an isotropic resolution of 18 μm and with a voltage setting of 70 kV. The resulting images were subsequently reconstructed into three-dimensional structures utilizing CTvox software. For the assessment of bone volume, CTan software (Bruker Version 1.15.4.0+) was employed; any dense masses located within soft tissue exhibiting Hounsfield units exceeding 272 were deemed indicative markers for HO.

### Cell culture, necroptosis, and osteogenic induction

Bone marrow-derived macrophages (BMDMs) were isolated from the bone marrow of C57BL/6 mice following established protocols.^6^ Briefly, the femur and tibia were excised, all soft tissue was carefully removed, and the bone marrow cavity was flushed with a 26 G needle to collect the bone marrow cells. The harvested cells were cultured in complete RPMI 1640 medium supplemented with 40 ng/mL of macrophage colony-stimulating factor (M-CSF). After an initial period of 3 days, the culture medium was refreshed, and the cells continued to incubate for an additional 3 days until they reached maturity. For experimental purposes, BMDMs were subsequently harvested and maintained in complete RPMI 1640 medium containing 40 ng/mL M-CSF (Peprotech, Cat# 315-02). Upon achieving a confluence of approximately 75%, BMDMs were primed with TNFα (50 ng/mL) and zVAD-fmk (50 μmol/L) to induce necroptosis.^63^

TSPCs were isolated from the Achilles tendon of C57BL/6 mice, following previously established protocols.^64^ The tendon tissue was finely dissected and subjected to enzymatic digestion using type I collagenase (Sigma-Aldrich, MO, USA) at 37 °C for a duration of 2 h. The resultant cell suspensions were cultured in a basic medium comprising α-MEM supplemented with 10% FBS, 100 U/mL penicillin, and 100 μg/mL streptomycin (all obtained from Gibco, USA) under conditions of incubation at 37 °C and an atmosphere of 5% CO_2_. To ensure optimal nutrient availability for the cells, the culture medium was refreshed every three days. Upon reaching confluence levels of ~80%–90% in the well plates, fully grown cells were harvested using trypsinization techniques and subsequently reseeded onto either 6-well or 24-well plates. Following this preparation phase, these cells were treated with an osteo-inductive medium that included FBS (10%), vitamin C (50 μg/mL), β-glycerophosphate disodium salt (10 mmol/L), and dexamethasone (0.01 μmol/L). Specific treatments were meticulously applied based on defined experimental groupings. WB analysis, IF staining of cells, alkaline phosphatase (ALP) and Alizarin red S (ARS) staining, proteomics sequencing, high-throughput whole transcriptome sequencing, seahorse tests, mass spectrometry, and co-immunoprecipitation (co‐IP) experiments were employed to assess alterations in cellular morphology and composition alongside uncovering the underlying mechanisms involved.

For the coculture assay, a transwell system (Corning, USA) featuring a porous membrane with a pore size of 0.4 µm was employed. BMDMs were seeded in the upper chamber of the transwell system, while TSPCs were cultured in the bottom chamber at a ratio of 4:1.

### EVs isolation and characterization

EV isolation techniques refer to our previously published article.^52^ EV-depleted serum was obtained through ultracentrifugation of FBS (Gibco, USA) at 100 000 × *g* for 18 h. The upper four-fifths of the supernatant were collected for further use. For the isolation of EVs, BMDMs were washed twice with PBS and subsequently incubated in RPMI 1640 medium supplemented with 10% EV-depleted serum for a duration of 48 h. Following this incubation period, supernatants from both PBS&DMSO-stimulated BMDMs and necroptotic BMDMs were harvested and subjected to centrifugation at 300 × *g* for 10 min. This was followed by a second centrifugation step at 2 000 × *g* for 20 min, and finally at 10 000 × *g* for an additional 30 min at a temperature of 4 °C to eliminate cells and cell debris. The resulting supernatants were then filtered through a sterile filter with a pore size of 0.22 µm before undergoing further ultracentrifugation at 120 000 × *g* for a total duration of 70 min at 4 °C; this process was repeated twice. The pelleted EVs were resuspended in PBS and stored at −80 °C until further analysis. The EVs isolated from PBS&DMSO-stimulated BMDMs and necroptotic BMDMs are referred to as ConMφ-EVs and NecroMφ-EVs, respectively.

The morphology of the EVs was examined using Transmission Electron Microscopy (TEM). The particle size distribution and concentration were characterized through Nanoparticle Tracking Analysis (NTA, ZetaView PMX 120, Particle Metrix, Meerbusch, Germany). WB analysis for Cd9, Cd63, Tsg101, Alix, F4/80, and Calnexin was also performed to confirm the successful isolation of EVs.^65^

Generally, EVs are derived from the endocytic processes of plasma membrane. The invagination of plasma membrane leads to the formation of early endosomes, characterized by marker like EEA1.^66^ Subsequently, these early endosomes undergo membrane budding to produce multivesicular bodies (MVBs), which can be identified by markers such as CD63. Upon fusion of the outer membrane of MVBs with cell membrane, vesicles are released into the extracellular space. Therefore, IF staining for Eea1 and Cd63 was employed to detect early and late stages of EV formation in BMDMs for target protein.

### Dil Labeling of EVs

To visualize the internalization of EVs, Dil dye was utilized for labeling. Isolated EVs were stained with a 2 µm working solution of Dil dye (Molecular Probes, USA) for a duration of 10 min. Following this incubation, excess Dil dye was eliminated through ultracentrifugation at 12 000 × *g* for 70 min. The labeled EVs were subsequently washed in PBS and subjected to another round of ultracentrifugation at 12 000 × *g* for 70 min to collect them, followed by filtration through a 0.22 µm membrane strainer.

An internalization assay was conducted by incubating TSPCs with Dil-labeled EVs for a period of 24 h. To stain the cytoskeleton, iFluor 488-conjugated phalloidin (Abcam, USA) was employed, while DAPI served as a counterstain. Images were captured using a fluorescence microscope (Eclipse TS100; Nikon Corporation, Tokyo, Japan).

### Proteome sequencing

Material for proteome analysis was obtained from NecroMφ-EVs and ConMφ-EVs. To redissolve the protein precipitate, 100 μL of a 50 mmol/L NH_4_HCO_3_ solution was added. Based on the protein amount, an appropriate volume of enzymolysis diluent was introduced (protein: enzyme ratio = 50:1 (m/m); for 100 μg of protein, add 2 μg of enzyme). The solutions were then incubated for digestion at 37 °C for either 12 h or overnight. The enzymatic reaction was halted by adjusting the pH to 3 through the addition of phosphoric acid. Subsequently, samples were desalted using SOLA™ SPE columns. After drying under vacuum, samples were resuspended with iRT peptides at a ratio of 1:10. The proteomic data analysis was conducted by Shanghai OE Biotech Co., Ltd. (Shanghai, China). All analyses utilized a Tims TOF Pro mass spectrometer (Thermo, Bruker) equipped with an Easyspray source (Thermo, USA). Samples were loaded onto a C18 column (15 cm × 75 µm) on an EASY-nLCTM 1200 system (Thermo, USA), with a flow rate set to 300 nL/min. A linear gradient was applied as follows: from minutes 0 to 20, 5% to 22% B; from minutes 20 to 24, 22% to 37% B; from minutes 24 to 27, 37% to 80% B; and final from minutes 27 to 30, 80% B. The ion mobility settings ranged between 0.7 and 1.3 Vs/cm², and collision energy was varied between 20 and 59 eV. The MS/MS spectra were recorded from 100 to 1 700 m/z. MS/MS spectra were searched using the Spectronaut Pulsar™ 18.4 (Biognosys, Swiss) against the Uniprot Mus Musculus database. Search database specific parameters included fixed modifications (Carbamidomethyl-C), variable modifications (oxidation-M and acetyl [protein N-term]), digestion (trypsin), a precursor Q-value cutoff (0.01), a protein Q-value cutoff (0.01), missed cleavage (2), and quantity MS-level (MS2).

### Cell immunofluorescence staining

Following treatment with various regimens, cells were fixed in 4% paraformaldehyde for 20 min and subsequently permeabilized using 0.5% Triton X-100 for 15 min. Next, the cells were blocked with BSA (1%) at room temperature for 1 h and then incubated overnight at 4 °C with antibodies against Runx2, Ocn, Opn, Pak4, Lcad, Mcad, p-Fabp3, and Fabp3. The following day, the cells were washed three times with PBS containing Tween20 (PBST) and stained with conjugated secondary antibodies under dark conditions. Nuclei were counterstained using DAPI (4,6‐diamidino‐2‐phenylindole; Beyotime, China), after which the cells were scanned employing a digital slide scanner (Pannoramic MIDI; 3DHISTECH Ltd).

### Real-time quantitative polymerase chain reaction (RT-qPCR)

Total RNA was extracted using either the Tissue RNA Purification Kit PLUS (EZBRN001-plus, EZBioscience, China) or the EZ-press RNA Purification Kit (B0004DP, EZBioscience) according to the manufacturer’s instructions. Following reverse transcription was conducted using the Color Reverse Transcription Kit RNA purification kit (A0010CGQ, EZBioscience) for complementary DNA synthesis. Quantification of the target gene expression was performed using the Color SYBR Green qPCR Master Mix (A0012-R2, EZBioscience). Primer sequences applied for the gene amplification are listed in Table S3, with *Gapdh* serving as the housekeeping gene.

### Alkaline phosphatase staining

TSPCs were washed three times with pre-cooled PBS and subsequently lysed on ice in pre-cooled 1% Triton X-100 for a duration of 30 min. The ALP activity of the cell lysate was assessed, with measurements taken at 405 nm normalized to the total protein concentration. Following this, TSPCs were washed another three times with PBS, fixed in 4% paraformaldehyde for 10 min, and then incubated with NBT-BCIP solution (Beyotime Biotech Company, Shanghai, China) for an additional 15 min. Subsequently, images were captured using an Olympus DP73 Microscope (Olympus, Tokyo, Japan). Finally, ALP activity was quantified utilizing an ALP assay kit (Nanjing Jiancheng Biotechnology Co., Ltd, China), adhering to the manufacturer’s instructions.

### Alizarin red S staining

The osteogenic differentiation of TSPCs was induced over a period of 21 days. Subsequently, the cells were thoroughly washed and fixed in 95% ethanol for 14 min. Following fixation, the cells were stained with a solution containing 2% ARS in Tris-HCl buffer at pH 4.3. Mineralized nodules were then observed using an inverted microscope. To elute the ARS staining, a solution of 10% cetylpyridine chloride was employed, and absorbance measurements were performed at a wavelength of 570 nm using a SpectraMax i3x spectrophotometer (Molecular Devices, Australia).

### Seahorse

The cells were inoculated with 100 μL of cell suspension per well in a 24-well cell metabolizer plate and allowed to adhere to the substrate before being placed into Seahorse XF Extracellular Flux Analyzers. Oxidative phosphorylation was evaluated by monitoring changes in the acidification rate and oxygen consumption rate.

### Mass spectrometry

The lysates of TSPCs samples, treated with various conditions, were prepared using the IP Lysis/Wash buffer and subjected to Co-IP utilizing the Pierce Direct Magnetic IP/CO-IP kit (#88828, Thermo Fisher Scientific), following the manufacturer’s instructions. Briefly, magnetic beads were initially conjugated with anti-Pak4 and Fabp3 antibodies or normal rabbit IgG. The resulting mixture was then incubated with the cell lysate on a rotator overnight at 4 °C. Subsequently, immunoprecipitants were eluted from the beads and combined with 5× loading buffer. Enzymatic digestion and peptide desalting were conducted sequentially prior to analyzing bound proteins via mass spectrometry using an Easy-nLC 1 200 Ultra High Performance Liquid Tandem Q Exactive High Resolution Mass Spectrometer. Co-expressed proteins among samples were identified through Venn plots.

### Co-immunoprecipitation

The boiled lysates from the aforementioned step were subsequently loaded onto a 4%-12% acrylamide gel and transferred to a nitrocellulose membrane. For the blocking process, we utilized the Protein-free rapid-blocking buffer (Epizyme). The membrane was then incubated overnight with the corresponding detection antibodies. On the following day, the membrane was treated with Clean-Blot IP detection reagent (#21230, Thermo Fisher Scientific) as a substitute for HRP-conjugated secondary antibodies. A chemiluminescence assay was conducted using chemiluminescence reagent (Epizyme, Shanghai), and imaging data were acquired using the ChemiDoc CRS imaging system (Bio-Rad, USA).

### Molecular modeling and docking analysis

The structures of murine Pak4 and Fabp3 were obtained from the RCSB Protein Data Bank (http://www.rcsb.org). Subsequently, the HDOCK software (http://hdock.phys.hust.edu.cn/) was utilized for docking experiments to generate the predicted protein binding modes. The resulting docking data were ultimately visualized using PyMOL and LigPlus software.

### Metabolites analyses

Metabolites analyses were performed by APTBIO Co (Shanghai, China). Mass spectrometry analyses for metabolites including palmitoyl-CoA and acetyl-CoA in cells were performed by Wuhan Greensword Creation Technology Co. Ltd. (Wuhan, China). Detailed protocols followed the methodology described by previous study.^67^

### Human samples collection

The research protocol (2022-KY-004) received approval from the Institutional Review Board at Shanghai Sixth People’s Hospital. Written informed consent was obtained and signed by either the patients themselves or their immediate family members. This study included healthy individuals aged 19 to 55 who developed posttraumatic HO following previous internal fixation surgeries for elbow fractures. Patients did not receive local radiotherapy treatment. Samples of HO were collected during subsequent elbow arthrolysis surgeries. All harvested HO samples in this study were at their maturation stage, in light of the ethical considerations that surgery was only performed once the HO had matured. Normal tendon served as control tissue for comparison with HO, as the latter originates from soft tissues, including tendons. Control samples were obtained from age-matched healthy individuals who underwent anterior cruciate ligament reconstruction surgery. The surplus normal hamstring tendon following its use for anterior cruciate ligament reconstruction was harvested for this purpose.

### Statistical analysis

The data analysis was conducted using GraphPad Prism 9 software. Data were presented as mean ± standard deviation (SD). Normality of the data was assessed using the Shapiro-Wilk test, and homogeneity of variance was evaluated using one-way ANOVA. Group comparisons were performed using independent *T*-test or one-way analysis of variance (ANOVA) followed by Tukey’s post hoc test for normally distributed data; otherwise, Kruskal–Wallis tests were used for non-normally distributed data. Statistical significance was defined as *P* < 0.05, and two-tailed tests were employed.

## Supplementary information

supplements

## Data Availability

The datasets used or analyzed during the current study are available from the corresponding author on reasonable request.
