# Supplementary material for: Pak4-mediated crosstalk between necroptotic macrophages and tendon stem/progenitor cells contributes to traumatic heterotopic ossification formation
Source: Bone Res. 2025 Oct 20;13:88. doi: 10.1038/s41413-025-00463-8 (PMC12536042; doi:10.1038/s41413-025-00463-8)
Supplement: Supplementary file 1 — supplements [file 41413_2025_463_MOESM1_ESM.docx]

**Supplementary Figure 1. Macrophage necroptosis was found in tendon lesions in burn/tenotomy mice at 3 weeks post-injury**


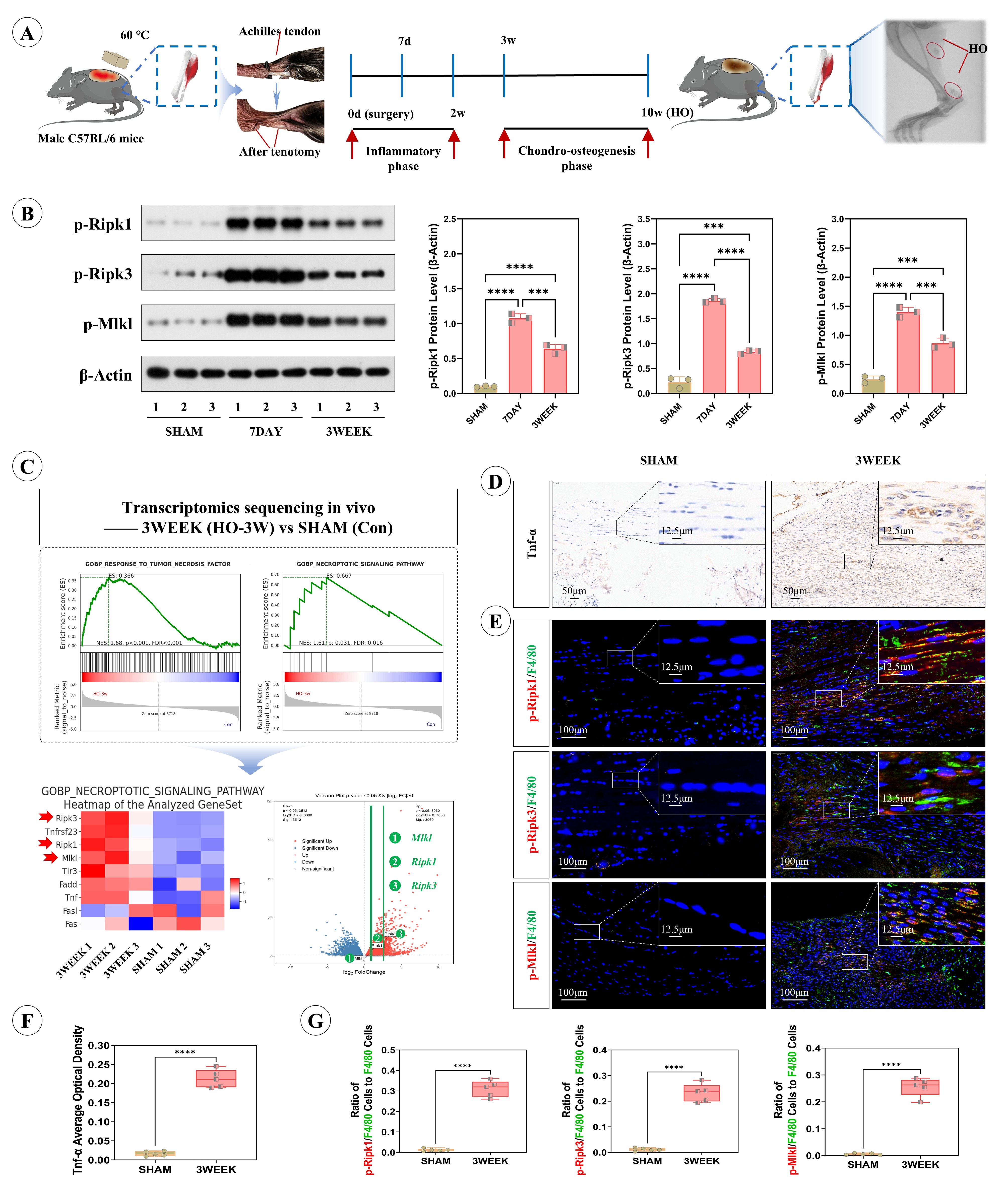


(A) Schematic depiction of the establishment of a murine burn/tenotomy model and different stages of HO progression.

(B) WB analysis was used to detect the levels of p-Ripk1, p-Ripk3 and p-Mlkl among the sham group and tendon lesions at 7 days and 3 weeks; N = 3, *** p < 0.001, **** p < 0.0001.

(C) High-throughput whole-transcriptome sequencing was performed and showed by GSEA for “response to tumor necrosis factor” and “necroptotic signaling pathway”, as well as heatmap and volcano plot for necroptotic indicators between sham group and the tendon lesions at 3 weeks; N = 3.

(D, F) IHC staining was used to detect the expression of Tnf-α in the sham group and tendon lesions at 3 weeks; N = 5, **** p < 0.0001, scale bar = 50 μm (original magnification) and 12.5 μm (insert magnification of the boxed area, 4.0x).

(E, G) IF staining was used to detect the positive cells of p-Ripk1, p-Ripk3 and p-Mlkl (red), co-localized with F4/80 (green), between the sham group and tendon lesions at 3 weeks; N = 5, **** p < 0.0001, scale bar = 100 μm (original magnification) and 12.5 μm (insert magnification, 4.0x).

**Supplementary Figure 2. Macrophage necroptosis contributed to aberrant osteogenic induction of TSPCs in vitro and traumatic HO formation in vivo**

**
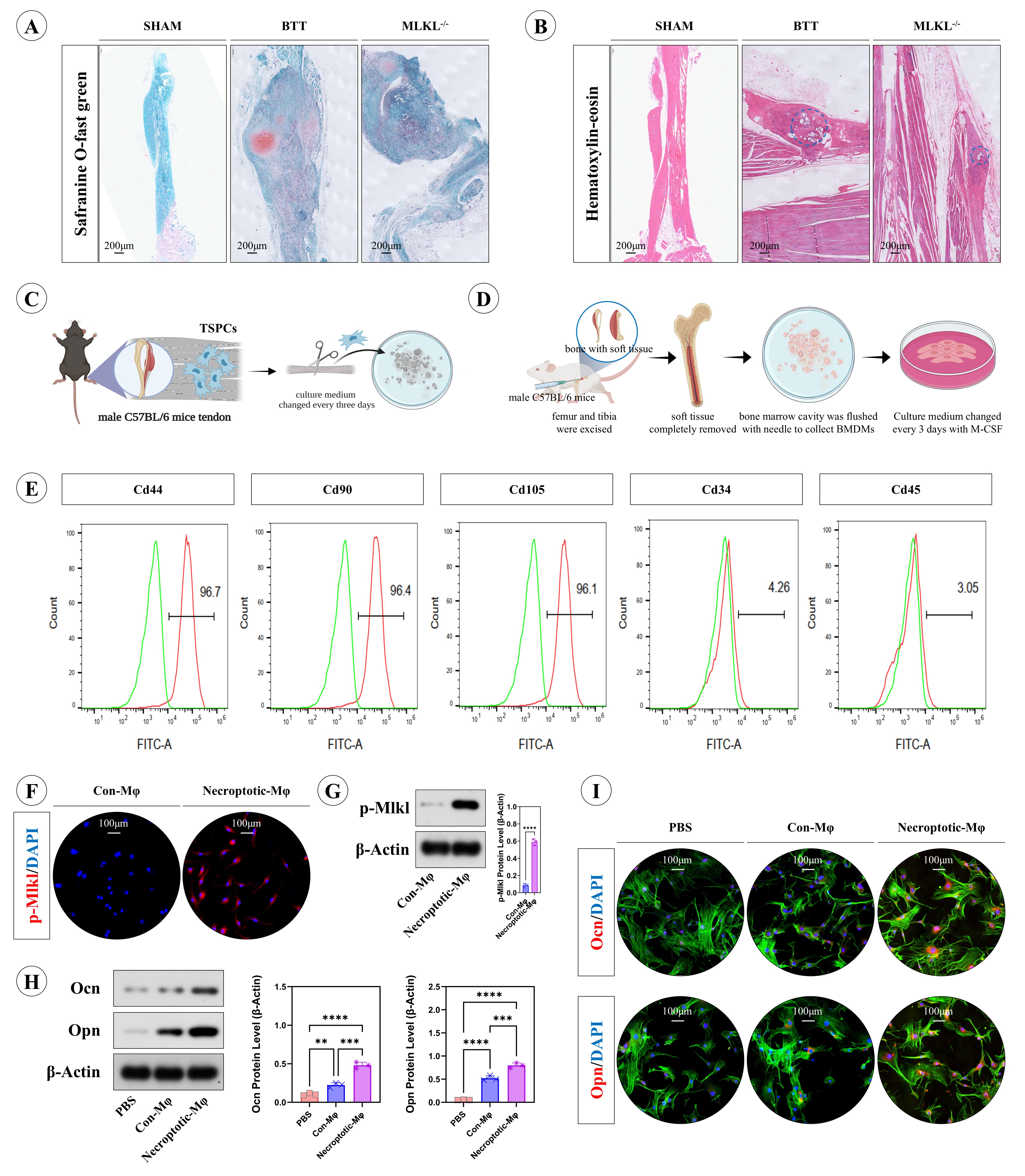
**

(A) SOFG staining was used to detect osteogenesis region among the sham group and tendon lesions at 3 weeks of WT mice and *Mlkl* KO mice; N = 5, scale bar = 200 μm (original magnification).

(B) H&E staining was used to detect ossification region among the sham group and tendon lesions at 10 weeks of WT mice and *Mlkl* KO mice; N = 5, scale bar = 200 μm (original magnification).

(C) Schematic depiction of the TSPCs isolation.

(D) Schematic depiction of the BMDMs isolation.

(E) Flow cytometry was used to characterize the MSC properties of TSPCs by identifying cell surface markers.

(F) IF staining was used to detect the expression of p-Mlkl (red), co-stained with DAPI (blue), in the necroptosis induced BMDMs; N = 6, scale bar = 100 μm.

(G) WB analysis was used to detect the expression of p-Mlkl in the necroptosis induced BMDMs; N = 3, **** p < 0.0001.

(H) WB analysis was used to detect the expression of Ocn and Opn in the osteogenic induced TSPCs in addition of PBS, Con-Mφ or Necroptotic-Mφ; N = 3, ** p < 0.01, *** p < 0.001, **** p < 0.0001.

(I) IF staining was used to detect the expression of Ocn and Opn (red), co-stained with phalloidin (green) and DAPI (blue), in the osteogenic induced TSPCs in addition of PBS, Con-Mφ or Necroptotic-Mφ; N = 6, scale bar = 100 μm.

**Supplementary Figure 3. Necroptotic macrophages derived EVs contributed to the osteogenic behavior of TSPCs in vitro and traumatic HO formation in vivo**

**
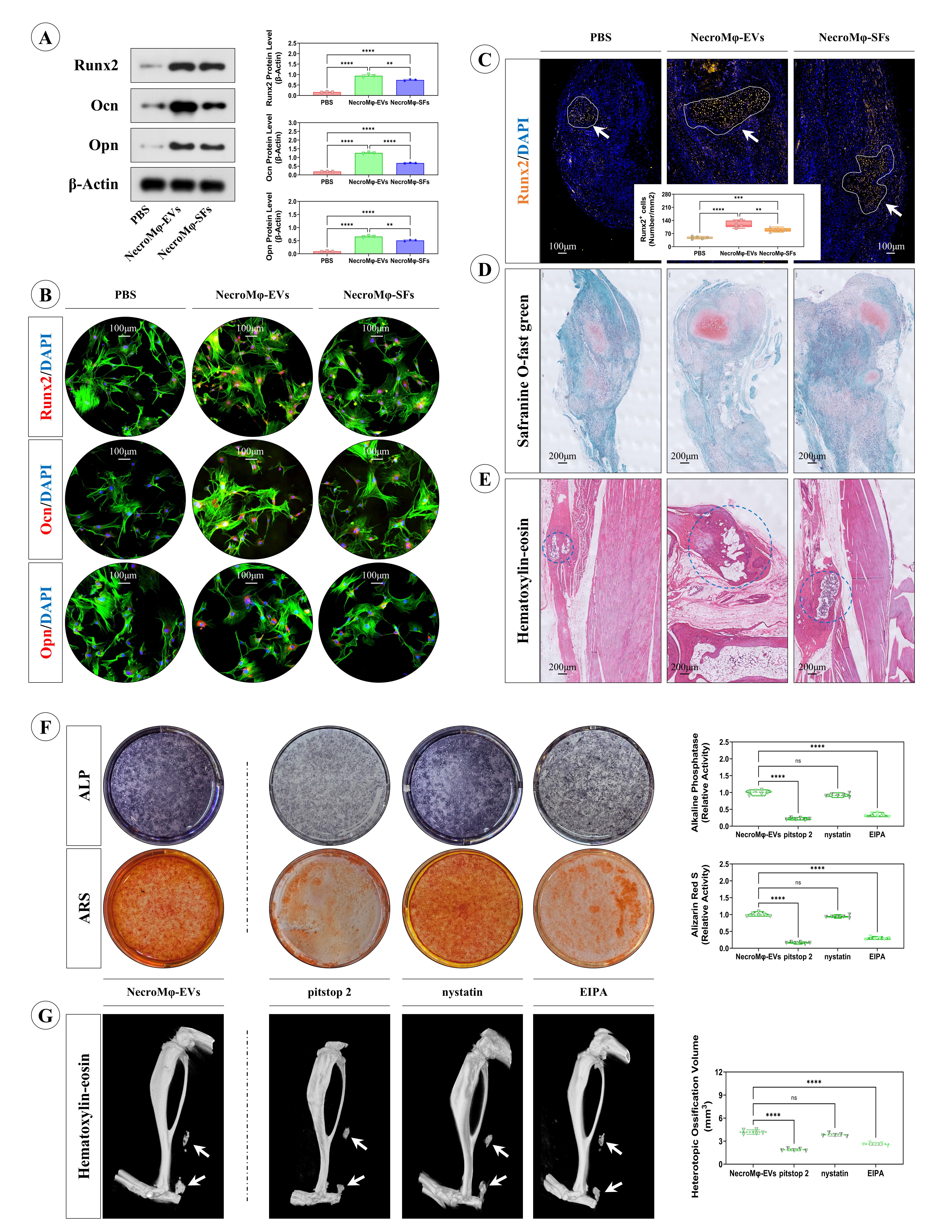
**

(A) WB analysis was used to detect the expression of Runx2, Ocn and Opn in the osteogenic induced TSPCs in addition of PBS, NecroMφ-EVs or NecroMφ-SFs; N = 3, ** p < 0.01, **** p < 0.0001.

(B) IF staining was used to detect the expression of Runx2, Ocn and Opn (red), co-stained with phalloidin (green) and DAPI (blue), in the osteogenic induced TSPCs in addition of PBS, NecroMφ-EVs or NecroMφ-SFs; N = 6, scale bar = 100 μm.

(C) IF staining was used to detect osteogenesis by the positive cells of Runx2 (orange) in the tendon lesions at 3 weeks in addition of PBS, NecroMφ-EVs or NecroMφ-SFs; N = 5, ** p < 0.01, *** p < 0.001, **** p < 0.0001, scale bar = 100 μm (original magnification).

(D) SOFG staining was used to detect osteogenesis region in the tendon lesions at 3 weeks in addition of PBS, NecroMφ-EVs or NecroMφ-SFs; N = 5, scale bar = 200 μm (original magnification).

(E) H&E staining was used to detect ossification region in the tendon lesions at 10 weeks in addition of PBS, NecroMφ-EVs or NecroMφ-SFs; N = 5, scale bar = 200 μm (original magnification).

(F) ALP and ARS staining were used to detect the osteogenesis of TSPCs in addition of NecroMφ-EVs, with or without pitstop 2, nystatin or EIPA; N = 6, **** p < 0.0001, ns, no significant difference.

(G) Micro-CT was used to detect HO formation in the tendon lesions at 10 weeks in addition of NecroMφ-EVs, with or without pitstop 2, nystatin or EIPA; the volume of HO was quantified from each group; N = 5, **** p < 0.0001, ns, no significant difference.

**Supplementary Figure 4. Necroptotic macrophages paracrine EVs incorporating Pak4 both in vitro and in vivo**

**
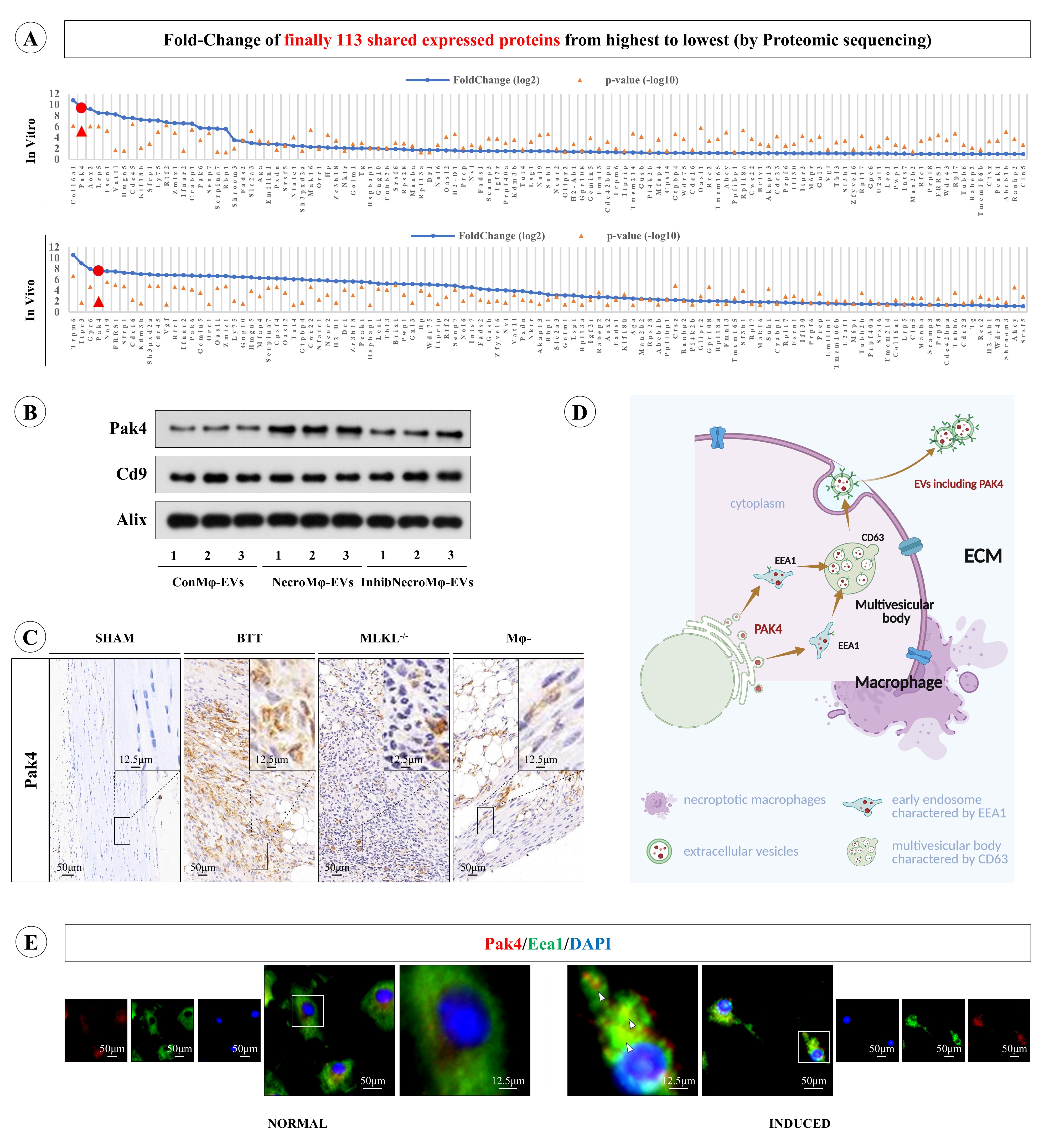
**

(A) All data of the finally 113 shared expressed proteins from highest to lowest fold-change by proteomic sequencing in vitro and in vivo for Figure 4A.

(B) WB analysis was used to detect the expression of Pak4 in EVs from BMDMs after indicated treatments; N = 3.

(C) IHC staining was used to detect the expression of Pak4 among the sham group and tendon lesions at 7 days after indicated treatments; N = 5, scale bar = 50 μm (original magnification) and 12.5 μm (insert magnification of the boxed area, 4.0x).

(D) Schematic depiction of incorporation of Pak4 into the EVs for trafficking upon macrophage necroptosis.

(E) IF staining was used to detect colocalization of Pak4 (red) and Eea1 (green) in BMDMs between normal group and necroptosis induction; N = 6, scale bar = 50 μm (original magnification) and 12.5 μm (insert magnification of the boxed area, 4.0x).

**Supplementary Figure 5. Pak4 from NecroMφ-EVs promoted osteogenic changes of TSPCs in vitro and traumatic HO formation in vivo**


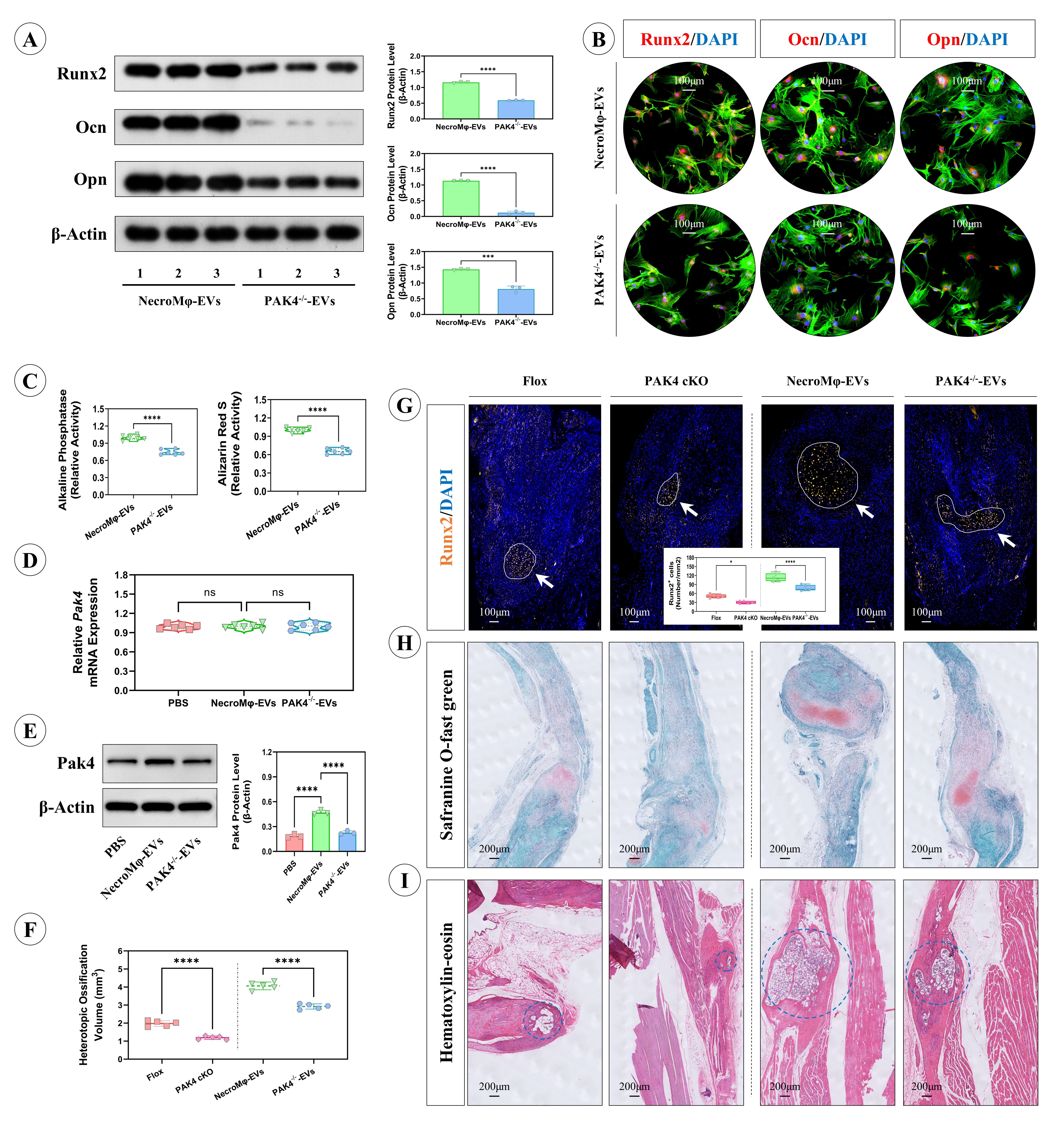


(A) WB analysis was used to detect the expression of Runx2, Ocn and Opn in the osteogenic induced TSPCs in addition of NecroMφ-EVs or PAK4^-/-^-EVs; N = 3, *** p < 0.001, **** p < 0.0001.

(B) IF staining was used to detect the expression of Runx2, Ocn and Opn (red), co-stained with phalloidin (green) and DAPI (blue), in the osteogenic induced TSPCs in addition of NecroMφ-EVs or PAK4^-/-^-EVs; N = 6, scale bar = 100 μm.

(C) Quantification for ALP and ARS staining in Figure 4D; N = 6, **** p < 0.0001.

(D) RT-qPCR was used to detect the transcription of *Pak4*, in the osteogenic induced TSPCs in addition of PBS, NecroMφ-EVs or PAK4^-/-^-EVs; N = 6, ns, no significant difference.

(E) WB analysis was used to detect the Pak4 protein concentration, in the osteogenic induced TSPCs in addition of PBS, NecroMφ-EVs or PAK4^-/-^-EVs; N = 3, **** p < 0.0001.

(F) Quantification for micro-CT in Figure 4E; N = 5, **** p < 0.0001.

(G) IF staining was used to detect osteogenesis by the positive cells of Runx2 (orange) between the tendon lesions at 3 weeks from Flox mice and *Pak4* cKO mice, as well as in addition of NecroMφ-EVs or PAK4^-/-^-EVs; N = 5, * p < 0.05, **** p < 0.0001, scale bar = 100 μm (original magnification).

(H) SOFG staining was used to detect osteogenesis region between the tendon lesions at 3 weeks from Flox mice and *Pak4* cKO mice, as well as in addition of NecroMφ-EVs or PAK4^-/-^-EVs; N = 5, scale bar = 200 μm (original magnification).

(I) H&E staining was used to detect ossification region between the tendon lesions at 10 weeks from Flox mice and *Pak4* cKO mice, as well as in addition of NecroMφ-EVs or PAK4^-/-^-EVs; N = 5, scale bar = 200 μm (original magnification).

**Supplementary Figure 6. Pak4 from NecroMφ-EVs reduced fatty acid β-oxidation (FAO) of TSPCs both in vitro and in vivo**

**
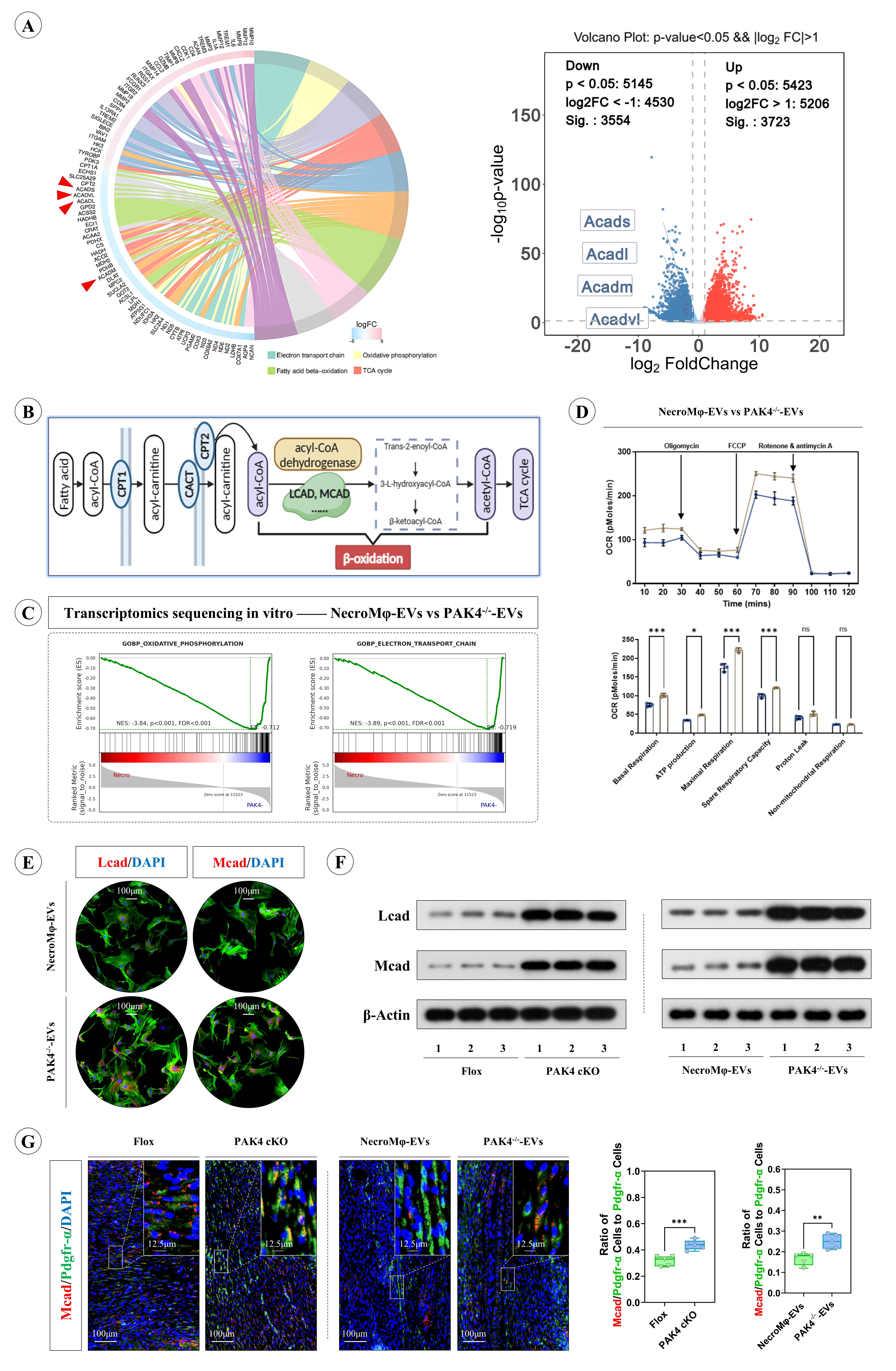
**

(A) High-throughput whole-transcriptome sequencing was performed and showed by chord diagram (top 10) and volcano plot, between the sham group and tendon lesions at 7 days; N = 3.

(B) Schematic depiction of the FAO process.

(C) High-throughput whole-transcriptome sequencing was performed and showed by GSEA for “oxidative phosphorylation” and “electron transport chain” between the osteogenic induced TSPCs in addition of NecroMφ-EVs or PAK4^-/-^-EVs in vitro.

(D) Seahorse test was used to detect the oxidative phosphorylation level in the osteogenic induced TSPCs in addition of NecroMφ-EVs or PAK4^-/-^-EVs; N = 3, ● represented NecroMφ-EVs group and ▲ represented PAK4^-/-^-EVs group, * p < 0.05, *** p < 0.001, ns, no significant difference.

(E) IF staining was used to detect the expression of Lcad and Mcad (red), co-stained with phalloidin (green) and DAPI (blue), in the osteogenic induced TSPCs in addition of NecroMφ-EVs or PAK4^-/-^-EVs; N = 6, scale bar = 100 μm.

(F) WB analysis was used to detect the levels of Lcad and Mcad between the tendon lesions at 7 days from Flox mice and *Pak4* cKO mice, as well as in addition of NecroMφ-EVs or PAK4^-/-^-EVs; N = 3.

(G) IF staining was used to detect the positive cells of Mcad (red), co-localized with Pdgfr-α (green), between the tendon lesions at 7 days from Flox mice and *Pak4* cKO mice, as well as in addition of NecroMφ-EVs or PAK4^-/-^-EVs; N = 5, ** p < 0.01, *** p < 0.001, scale bar = 100 μm (original magnification) and 12.5 μm (insert magnification of the boxed area, 4.0x).

**Supplementary Figure 7. Pak4 from NecroMφ-EVs promoted the osteogenic behavior of TSPCs in vitro and traumatic HO formation in vivo by downregulating fatty acid β-oxidation (FAO)**

**
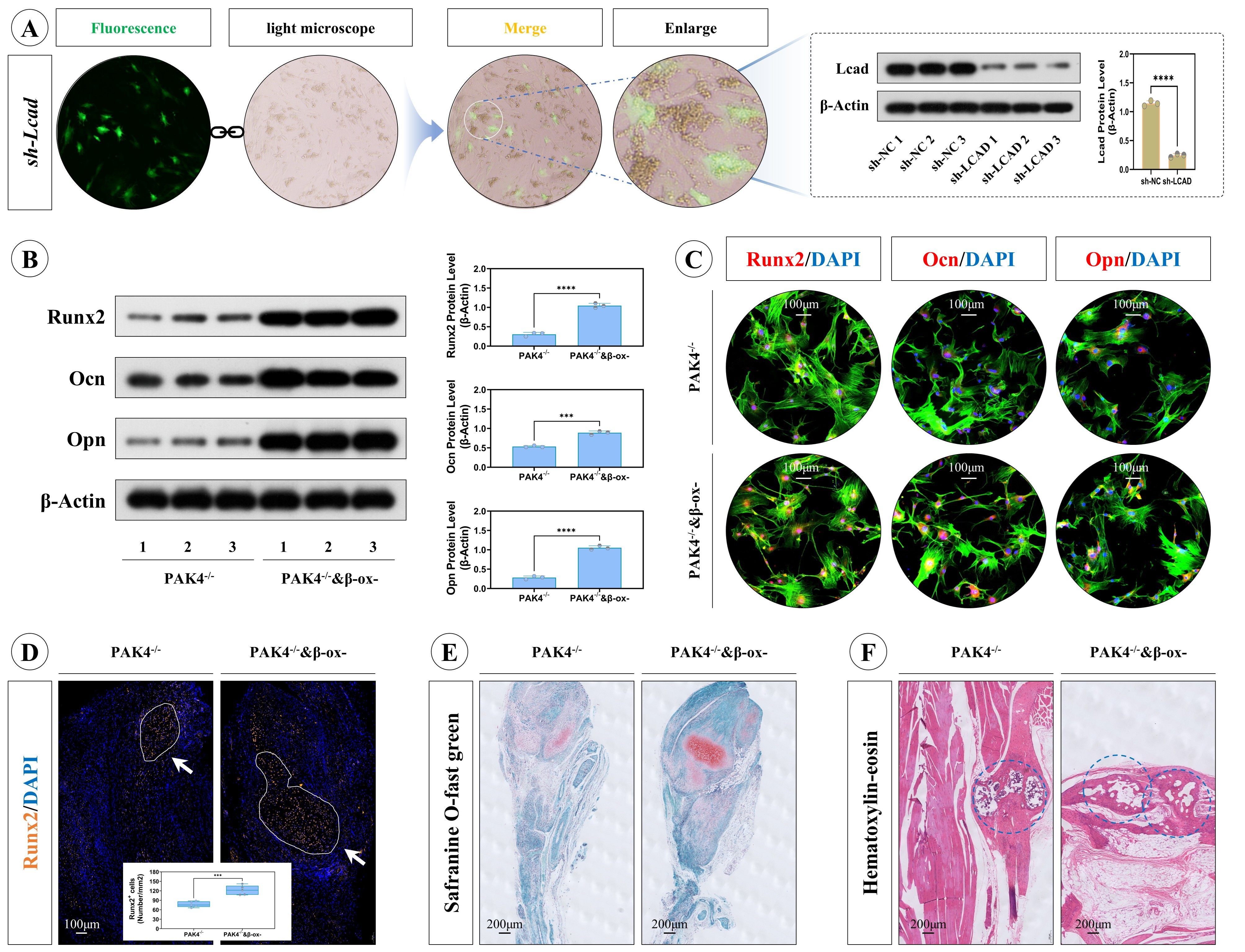
**

(A) Fluorescence and light microscope and WB analysis were used to confirm success of downregulation of *Lcad* transfection for TSPCs. N =3; **** p < 0.0001.

(B) WB analysis was used to detect the expression of Runx2, Ocn and Opn in the osteogenic induced TSPCs in addition of PAK4^-/-^-EVs, with or without *sh-Lcad*; N = 3, *** p < 0.001, **** p < 0.0001.

(C) IF staining was used to detect the expression of Runx2, Ocn and Opn (red), co-stained with phalloidin (green) and DAPI (blue), in the osteogenic induced TSPCs in addition of PAK4^-/-^-EVs, with or without *sh-Lcad*; N = 6, scale bar = 100 μm.

(D) IF staining was used to detect osteogenesis by the positive cells of Runx2 (orange) in the tendon lesions at 3 weeks in addition of PAK4^-/-^-EVs, with or without *sh-Lcad*; N = 5, *** p < 0.001, scale bar = 100 μm (original magnification).

(E) SOFG staining was used to detect osteogenesis region in the tendon lesions at 3 weeks in addition of PAK4^-/-^-EVs, with or without *sh-Lcad*; N = 5, scale bar = 200 μm (original magnification).

(F) H&E staining was used to detect ossification region in the tendon lesions at 10 weeks in addition of PAK4^-/-^-EVs, with or without *sh-Lcad*; N = 5, scale bar = 200 μm (original magnification).

**Supplementary Figure 8. Pak4 from NecroMφ-EVs directly binds to Fabp3 at S122 phosphorylation site in TSPCs in burn/tenotomy mice**


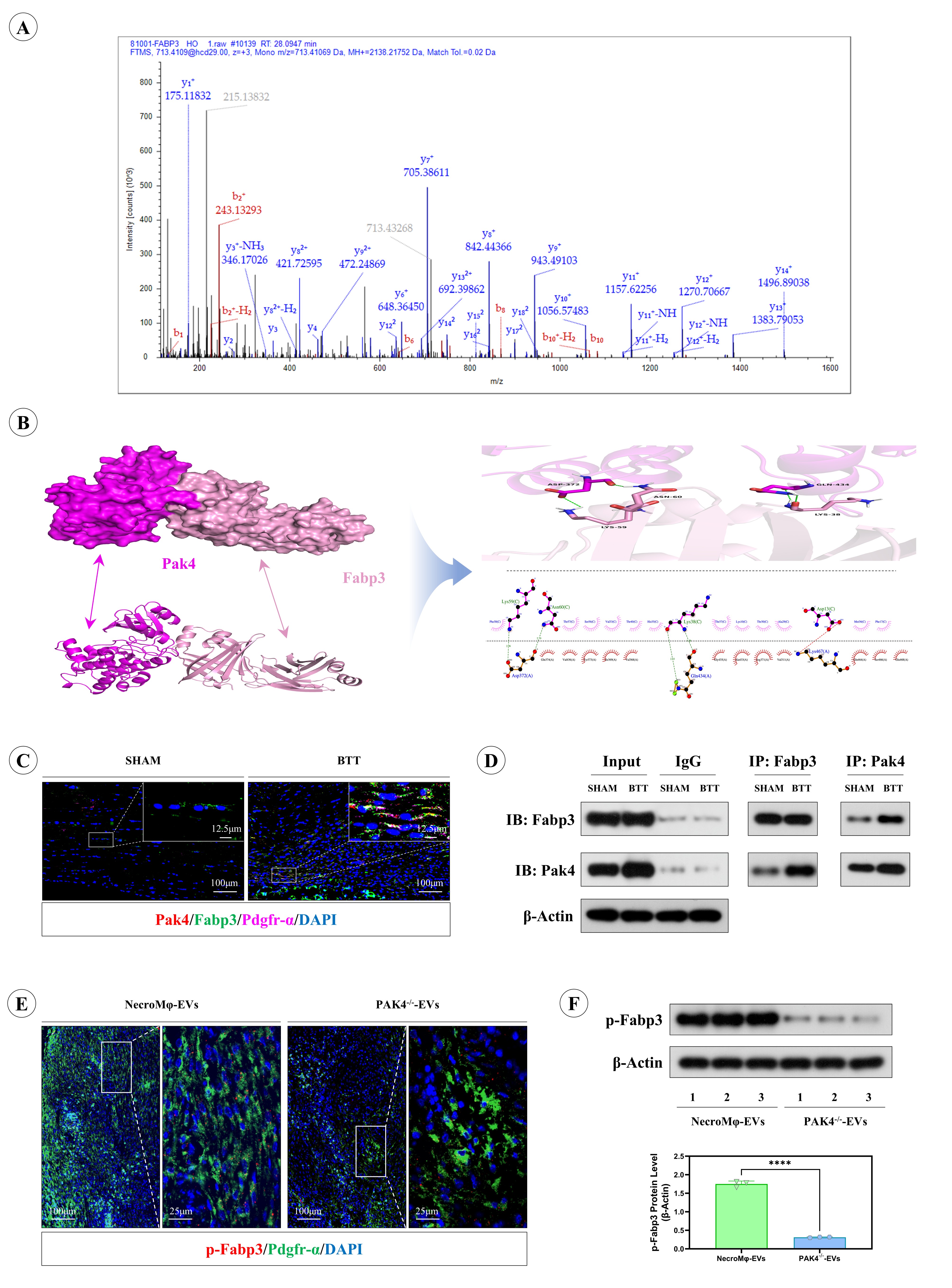


(A) Mass spectrometry analysis was used to detect the potential molecules that could bind with Pak4 between the sham group and tendon lesions at 7 days; N=3. Results of Fabp3 were showed.

(B) Molecular modeling and docking analysis were used to predict the physical binding ability between murine Pak4 and Fabp3.

(C) IF staining was used to detect colocalization of Pak4 (red) and Fabp3 (green), co-localized with Pdgfr-α (purple), between sham group and the tendon lesions at 7 days; N = 5, scale bar = 100 μm (original magnification) and 12.5 μm (insert magnification of the boxed area, 4.0x).

(D) Co-IP analysis was used to verified the bind relationship between Pak4 and Fabp3 in the tendon lesions at 7 days and sham group; N=3.

(E) IF staining was used to detect colocalization of p-Fabp3 (red), co-localized with Pdgfr-α (green), in the tendon lesions at 7 days in addition of NecroMφ-EVs or PAK4^-/-^-EVs; N = 5, scale bar = 100 μm (original magnification) and 25 μm (insert magnification of the boxed area, 4.0x).

(F) WB analysis was used to detect the expression of p-Fabp3 in the tendon lesions at 7 days in addition of NecroMφ-EVs or PAK4^-/-^-EVs; N = 3, **** p < 0.0001.

**Supplementary Figure 9. Phosphomimetic mutant of FABP3 on S122 site increased the osteogenic behavior of TSPCs in vitro**


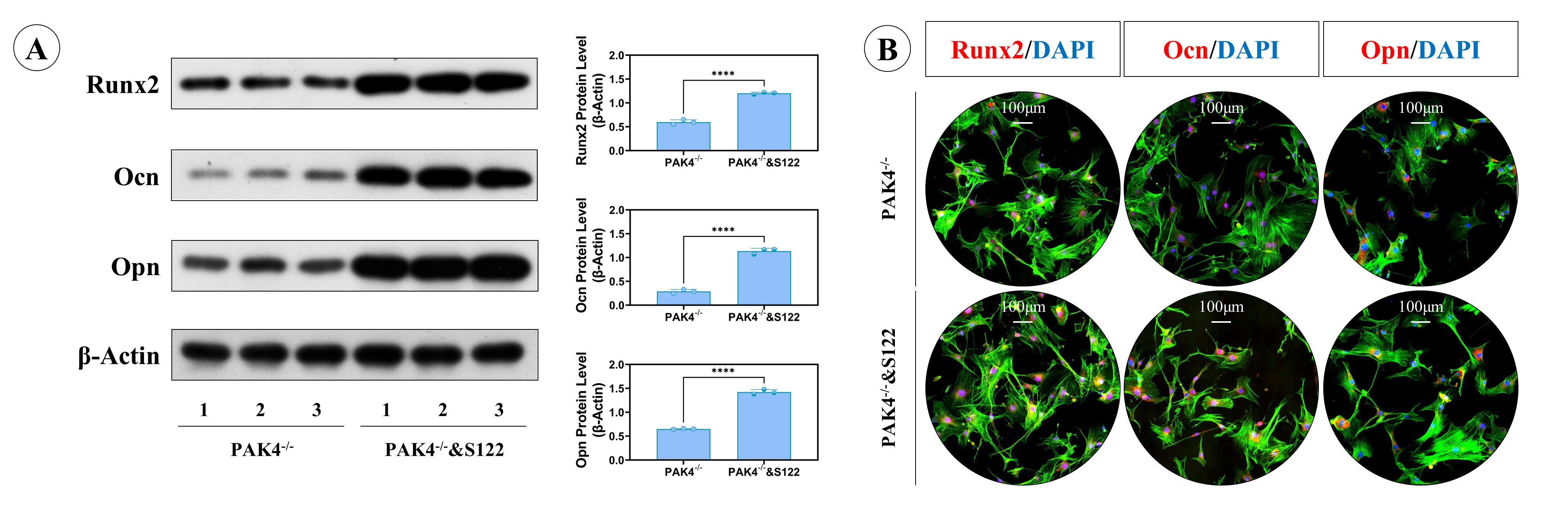


(A) WB analysis was used to detect the expression of Runx2, Ocn and Opn in the osteogenic induced TSPCs in addition of PAK4^-/-^-EVs, with or without *Fabp3^S122D^*; N = 3, **** p < 0.0001.

(B) IF staining was used to detect the expression of Runx2, Ocn and Opn (red), co-stained with phalloidin (green) and DAPI (blue), in the osteogenic induced TSPCs in addition of PAK4^-/-^-EVs, with or without *Fabp3^S122D^*; N = 6, scale bar = 100 μm.

**Supplementary Figure 10. The field of interest for immunohistochemical and immunofluorescence staining**


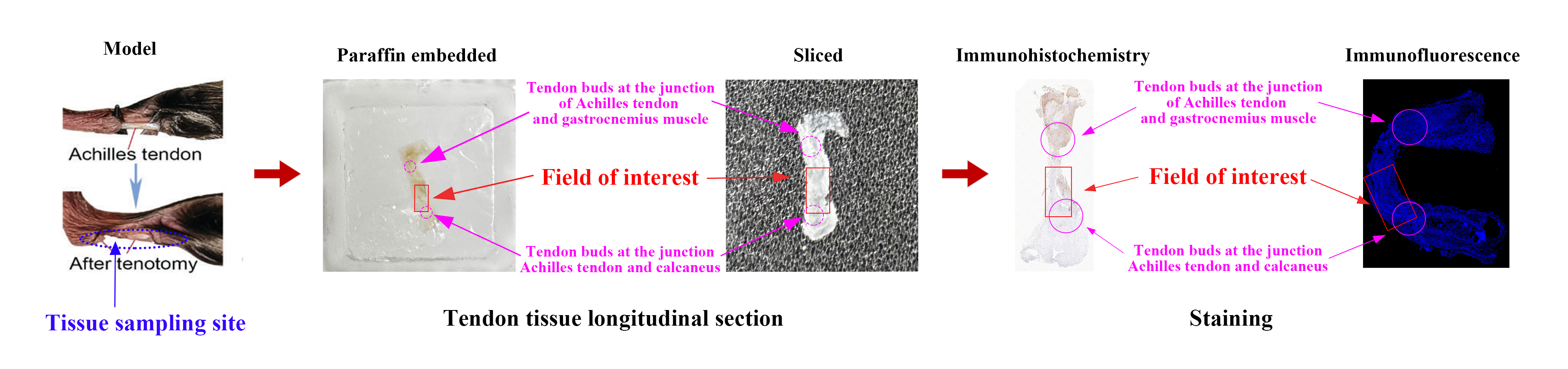


**Supplementary Table 1.** Primers used for genotyping the *Mlkl^-/-^* mice.

| PCR No. | Primer No. | Sequence | Band Size |
| --- | --- | --- | --- |
| PCR① | T002470-F1 | TGGGCATATAGATGATCTTGGACAC | WT:1949bp  KO:270bp |
|  | T002470-R1 | AACCTGTGATTCAACATTTCCAAT |  |
| PCR② | T002470-F1 | TGGGCATATAGATGATCTTGGACAC | WT:431bp  KO:0bp |
|  | T002470-R2 | GCCTCCTTTTCCAATCTTGCCT |  |

**Supplementary Table 2.** Primers used for genotyping the *Lyz2-cre::Pak4^flox/flox^* mice.

| PCR No. | Primer No. | Sequence | Band Size |
| --- | --- | --- | --- |
| ①(5'arm) | T018538(P1)-F1 | GCAGTGGATGCCGGAAAGAAA | WT:388bp  Targeted:493bp |
|  | T018538(P1)-R1 | ACCAGGTCTTGGACCTAGATCAGAGTC |  |
| ②(3'arm) | T018538(P1)-F2 | GGTGGAACTGGGACCCTTTA | WT:261bp  Targeted:295bp |
|  | T018538(P1)-R2 | CTGTTCCTCGTTCATCCTGC |  |

**Supplementary Table 3.** Primers used for qRT-PCR.

| Genes | Forward | Reverse |
| --- | --- | --- |
| Murine *Pak4* | GGGTCTGCGTGCTGATTCCTCAAT | CGGATCATCTTCATAGCTTTGAGGGG |
| Murine *Gapdh* | CCTCGTCCCGTAGACAAAATG | TGAGGTCAATGAAGGGGTCGT |
